# Connected to Others and Kind to Self: Social Connection and Self‐Affiliation Change in Open‐Ended Psychotherapy

**DOI:** 10.1002/jclp.70220

**Published:** 2026-09-04

**Authors:** Andreas Voldstad, Ole André Solbakken, Jesús Montero‐Marín, Jon Trygve Monsen, Erkki Heinonen

**Affiliations:** ^1^ Department of Psychiatry University of Oxford Oxford UK; ^2^ Department of Psychology Faculty of Social Sciences, University of Oslo Oslo Norway; ^3^ Teaching, Research & Innovation Unit, Parc Sanitari Sant Joan de Déu Sant Boi de Llobregat, Barcelona Spain; ^4^ Consortium for Biomedical Research in Epidemiology and Public Health (CIBER Epidemiology and Public Health—CIBERESP) Madrid Spain; ^5^ Finnish Institute for Health and Welfare Helsinki Finland

**Keywords:** cohort study, longitudinal, psychotherapy, self‐relatedness, social connection, social support

## Abstract

**Objective:**

Social connection predicts health, but remains largely underexplored as an outcome of psychotherapy. A friendly relationship to oneself is linked to positive relationships to others, but it is not clear how they influence each other across time during and after treatment. This study evaluates change in a measure of social connection and support, the Quantity and Quality of Social Relations (QQSR), and investigates its longitudinal associations with the Structural Analysis of Social Behavior Introject self‐affiliation.

**Methods:**

370 adult outpatients in Norway with various presenting problems were assessed before and after open‐ended psychotherapy, with up to 2.5 years follow‐up. We fitted latent growth curves and structured residual models.

**Results:**

There was significant growth in QQSR across long‐term follow‐up, particularly in support and non‐family relationships. There was no change from pre‐ to posttreatment, and significant between‐patient variation in both intercepts and growth rates. Improved QQSR after treatment predicted improved self‐affiliation in the next follow‐up, which in turn predicted improved QQSR. These effects were robust to detrending and controlling for pretreatment personality disorder. Pretreatment QQSR correlated with self‐affiliation growth.

**Conclusion:**

On average over a long timespan, patients' social connection shows gradual positive growth, and reciprocal lagged associations with self‐affiliation. The QQSR might measure relatively stable characteristics and between‐patient differences relevant to self‐affiliation change. Clinicians might screen for social and self‐relationships throughout therapy, both as outcomes and resources.

## 1 Introduction

Social connection includes *structure*, e.g., the presence of other people in one's life and frequency of contact, *function*, e.g., perceived support, and *quality*, e.g., affective aspects of relationships (Holt‐Lunstad 2021). Across various operationalizations, social connection shows a substantial meta‐analytic association with health and longevity (Albasheer et al. 2024; Holt‐Lunstad et al. 2010; Meng et al. 2024; Wang et al. 2023). Social connection also predicts mental health (Morina et al. 2021; Wang et al. 2018; Wickramaratne et al. 2022), although more prospective research is needed (Pearce et al. 2023). In addition, social connection might be considered an important outcome in its own right, beyond its association with other outcomes (Doyle and Link 2024). Nevertheless, the protective effect and intrinsic value of relational outcomes have arguably been underappreciated in healthcare practice and outcome research (Holt‐Lunstad 2021).

To our knowledge, only a few studies have evaluated social connection outcomes of psychotherapy, particularly social support. A meta‐analysis of 11 trials of psychological treatment for depression found a significant small‐to‐medium effect on social support (Park et al. 2014). Most studies included short‐term treatments without long‐term follow‐up. In a recent naturalistic study of 326 adult outpatients suffering from depression and/or anxiety, randomized to solution‐focused, short‐term or long‐term psychodynamic therapy, all treatment groups showed significant improvement in social support (Laurila et al. 2024). However, there is a lack of research on social connection and support in open‐ended psychotherapy.

Various psychological theories posit that social connection contributes to satisfying fundamental needs essential for mental health, well‐being, and functioning (Andersen et al. 2021). In social provisions theory, different categories of relationships typically provide for different social connection needs, including intimacy, often found in partner relationships, belonging, often found with friends, nurturing others, often found with one's children, competence, often found with work colleagues, available support, often found with kin, and guidance (Mancini and Blieszner 1992). Indeed, a recent study suggests that interacting with diverse relationship types predicts well‐being over and beyond the amount of social interaction (Collins et al. 2022). In addition, some researchers propose humans have a need for giving and receiving affection, defined as communicating liking, care, and concern across “relationships ranging from acquaintance to intimate” and characterizing affection as a form of social support (Pendell 2002, p. 70). Similarly, the need to belong theory posits that frequent positive interactions in enduring relationships characterized by mutual affection and caring are necessary to satisfy a fundamental need to belong (Baumeister and Leary 1995). Given and received affection are associated with mental health and well‐being (Floyd 2019), but are often not included in measures of social connection and support.

As social connection is central to our health and well‐being, its investigation in psychotherapy research opens a promising avenue for understanding how treatment may lead to meaningful improvements beyond symptom change. In this study, we investigate a secondary outcome of the Norwegian Multisite Study of Process and Outcome in Psychotherapy (NMSPOP) (Havik et al. 1995; Nordmo 2018) which has not yet been evaluated as a treatment outcome. The Quantity and Quality of Social Relations (QQSR) is a measure of social connection. The designers of the NMSPOP developed the QQSR because they were not aware of a comparable instrument at the time to assess the breadth, frequency of contact, and quality of a patient's social network in specific relationship categories. It was based on social provisions theory, the need to belong, and clinical experience, focusing on what the designers of NMSPOP deemed to be important aspects of social connection. The QQSR is similar to the Network of Relationships Inventory (NRI) available at the time (Furman and Buhrmester 2009), where patients answer 30 items for each of several members of their social network, yielding 10 scales for each person they rate, including various forms of social support and negative interactions. Like the NRI, the QQSR yields a matrix of relationships by relationship qualities. However, the QQSR has fewer items and includes quantity. It was designed for easy administration in a clinical setting, giving a one‐page overview of a patient's prototypical social network. The QQSR includes four *relationship aspects*: frequency of contact, given and received affection, and helpful contact in times of difficulty, and 10 different *relationship categories*, including family of origin, partner and child, and non‐family relationships. In terms of Holt‐Lunstad's broad definition, it operationalises social connection *structure* in the presence of typical social relationships and their frequency of contact, *function* as perceived emotional support, and *quality* as the degree of expressed and received affection in the relationship.

Patients who seek psychotherapy often suffer from both distressing relationships to others and a hostile relationship to themselves, both of which are linked to more psychopathology (Bjerke et al. 2016; Neff 2023). Various theories suggest links between how we relate to ourselves and how we relate to others. People may act out self‐to‐self relationship behaviors similar to self‐to‐other behaviors, such as being critical, controlling, or kind to oneself (Gilbert et al. 2004). According to attachment theory, people internalize ways of treating themselves based on how they have been treated by others, and conversely their self‐image affects how they experience relationships with others (Shaver and Mikulincer 2016). This forms the theoretical basis for the introject scales of the Structural Analysis of Social Behavior (SASB‐I), operationalising kind behavior towards oneself along the dimension of self‐affiliation versus hostility (Critchfield and Benjamin 2024). In two large naturalistic datasets, NMSPOP and the Helsinki Psychotherapy Study, patients changed to a less hostile and more loving self‐relationship from pre‐ to posttreatment (Halvorsen and Monsen 2007; Lindfors et al. 2015). However, it is not clear how social connection and self‐affiliation are related to each other over time. For example, psychotherapy might help patients improve their social connection, and thereby influence how they relate to themselves. Conversely, psychotherapy might help patients improve their relationship to themselves and thereby make it easier to connect with others. Psychotherapy itself is a relational experience that may impact self‐ and other relationships (Wampold and Imel 2015).

In this exploratory study, we examine relationships to self and others in a sample of patients suffering from various mental disorders, including personality disorders, receiving open‐ended psychotherapy in a naturalistic outpatient setting. We investigate the following research questions: (1) Does social connection change from pretreatment across follow‐up? We hypothesize positive growth in social connection from pretreatment to first follow‐up after treatment, and continued growth across long‐term follow‐up. Our primary outcome is a global index of social connection. As secondary outcomes, we explore which relationship aspects and categories in particular show evidence of change. (2) Do self‐affiliation and social connection predict each other across time? We hypothesize positive cross‐lagged effects. In particular, we hypothesize that self‐affiliation will be a “driver” of change in social connection.

## 2 Materials and Methods

### 2.1 Patients and Setting

NMSPOP was a naturalistic longitudinal study of 370 adult patients undergoing psychotherapy treatment at 8 outpatient sites in Norwegian public healthcare between 1995 and 2008. Exclusion criteria were age below 20, IQ below 70, serious substance use disorder, psychosis, and need for emergency treatment or hospitalization. The Regional Committee for Medical Research Ethics in Eastern Norway approved the study (approval number S‐95010). After receiving information, patients signed a written consent. Patients were not compensated for participation. The mean age was 34.9 (SD = 9.4) with 71% female patients. About half the sample (54%) were married or cohabiting. The mean number of children was 1.5 (SD = 1.2). About a third (38%) had higher education. Patients had on average 3.1 (SD = 1.5) siblings. About half (56%) were occupationally functioning. Around half (53%) had a personality disorder (PD); the most common were depressive (*n* = 102), avoidant (*n* = 98) and obsessive (*n* = 54). The most common Axis I disorders were major depression (*n* = 117), social phobia (*n* = 97), and generalized anxiety disorder (*n* = 82). See Supplement S1 for complete descriptives.

The 88 therapists were instructed to provide their usual therapeutic practice. Treatment was conducted by qualified healthcare professionals with specific psychotherapy training, including clinical psychologists, psychiatrists, psychiatrists in training, psychiatric nurses, or clinical social workers. Mean therapist experience was 10 years (SD = 6.57). The most common patient caseload was five patients (range = 1–10). The majority of therapists (78%) reported being influenced by a psychodynamic orientation. Some therapists also reported being influenced by humanistic therapies (30%) and/or cognitive behavioral therapies (30%). The mean number of sessions was 51.3 (SD = 58.9, median = 35). Treatment was open‐ended, with the exception of one treatment site where treatment was limited to 40 sessions. Therapists and patients were instructed to agree when to terminate treatment based on their joint appraisal of progress.

### 2.2 Assessments and Measures

Outcome assessments were conducted in up to six waves: T1: pretreatment, T2: first follow‐up after treatment completion, T3: 6 months after T2, T4: 1 year after T2, T5: 2 and a half years after T2, T6: 6 years after T2. Mean treatment length was 20.6 months (SD = 19.7, median = 13.8). Mean delay between treatment completion and T2 assessment was 9.8 months (SD = 25.5, median = 2.46). Of 370 patients, 367 responded to the QQSR at T1, 293 at T2, 223 at T3, 230 at T4, 256 at T5, and 23 at T6. For self‐relatedness, 366 responded to the SASB‐I at T1, 289 at T2, 223 at T3, 231 at T4, 255 at T5, and 23 at T6.

In the *Quantity and Quality of Social Relations* (QQSR), patients first indicate whether they have a spouse or cohabiting partner, non‐cohabiting romantic partner, daughter, son, mother, father, sister, brother, other family member, male friend, female friend, neighbors, and work colleagues. In the current study, this was simplified to romantic partner, child, mother, father, sibling, other family member, male friend, female friend, neighbors, and work colleagues, using the contact they see most frequently to collapse categories such as sibling and child. Patients then responded to four items for each relationship they have: the frequency of contact (scored 1–6, where 1 indicates having no contact, 2 indicates contact four times a year or less, 3 indicates monthly contact, 4 indicates fortnightly contact, 5 indicates weekly contact, and 6 indicates daily contact), *freely expressed affection*: “How affectionate can you be towards these persons, i.e., how freely can you show them that you care about them?” and *received affection*: “How affectionate do you perceive the person(s) can be towards you?” (scored 1–5, where 1 indicates “to a small degree” and 5 indicates “to a large degree”), and *support*: “When you have problems or experience something difficult, do you have contact with any of these persons, and do you perceive it as helpful?” (scored from 1 to 4, where 1 indicates “No,” 2 indicates “Yes, but no help,” 3 indicates “Yes, some help,” and 4 indicates “Yes, much help”). If there are multiple persons in a category, patients are instructed to respond regarding the person they see most often.

The relationship by aspect structure of the QQSR offers flexibility in how to aggregate it. We opted to summarize the QQSR in formative indexes based on the conceptual unity of the items, so that mean scores across various relationships form the patient's social connection. In contrast to scale items, the items forming an index need not covary. Therefore, DeVellis and Thorpe (2023, p. 219) argue it may be illogical to evaluate internal consistency of formative indexes. However, we acknowledge that it could be equally valid to view the QQSR indexes as scales. We therefore report internal consistencies (Cronbach's alpha assessed at T1) below.

Consistent with the intended use of the QQSR, we first scored a global mean index (QQSR total, *α* = 0.90), and separate indexes for each relationship aspect scale: Contact (*α* = 0.68), freely expressed affection (*α* = 0.80), received affection (*α* = 0.78), and Support (*α* = 0.76) (Hersoug et al. 2002; Høglend et al. 2007). We also scored indexes representing groups of relationship categories that are consistent with related measures of social support (Cohen 1997; Lindfors et al. 2014; Marijuán et al. 2017; Zimet et al. 1988), namely: current family (*α* = 0.64), family of origin (*α* = 0.90) and outside family (*α* = 0.86). To disentangle the presence of relationships from their aspects, missing relationships were ignored when creating indexes, and we calculated separate indexes for the number of relations present in the patient's life (*α* for these “*N* relationships” indexes ranged from 0.31 to 0.45 and are reported in Supplement S1). This scoring allowed us to reduce the QQSR to a manageable number of meaningful indexes that maintain the granularity of distinguishing between different relationships and aspects of social connection. The low internal consistencies of the “*N* relationships” indexes suggest they are best perceived as summary indexes of a patient's relationship categories. By contrast, there appears to be a high degree of consistency between the relationships and relationship aspects forming the other indexes.

Before proceeding, we examined convergent validity of the QQSR indexes at T1, using the rich set of other outcome variables available in the NMSPOP. The QQSR indexes showed a pattern of negative association with clinician‐assessed personality pathology, and self‐report interpersonal problems and psychiatric symptoms, and positive association with clinician‐rated global functioning, relational functioning, affect consciousness, and self‐reported self‐affiliation, parental warmth, and early working alliance. These bivariate correlations are reported in Supplement S2. Continuous QQSR pretreatment scores were approximately normal, except for potential ceiling effects in the current family index.

*The Structural Analysis of Social Behavior—Introject Surface* (SASB‐I) contains 36 items describing how a person treats themselves. It consists of eight subscales or “clusters” along a circumplex based on the orthogonal dimensions self‐autonomy and self‐affiliation (Benjamin 1996). Patients were instructed to rate “yourself as you usually are.” Items were rated from 0 (never) to 10 (always). Cluster scores were calculated as mean scores. Self‐affiliation (Saff) was then calculated as weighted sums of the clusters with reference to their position in the circumplex (see Pincus et al. 1998). Higher scores indicate more loving, and lower scores more attacking, self‐directed behavior.

### 2.3 Statistical Methods

All analyses were implemented in R (version 4.4.3) using *lavaan* (Rosseel 2012). Except for descriptives, we excluded T6 due to sparse data. To evaluate whether social connection changed, we tested the effect of time from T1 to T5 and T1 to T2 in univariate unconditional latent curve models (LCMs), using the repeated measures as indicators of intercept and slope growth factors (Curran et al. 2014). For T1–T5, years from baseline (T1) were chosen as the fixed occasion time estimate. Median treatment time and median delay from treatment end to first posttreatment assessment were added to code time for T2. For T1–T2, we estimated a fixed slope with random intercepts (i.e., the variance of the slope was fixed to 0). To account for testing multiple versions of the QQSR outcome, we controlled the False Discovery Rate (FDR) by applying the Benjamini‐Hochberg (BH) procedure (Benjamini and Hochberg 1995) to a vector of all *p*‐values (i.e., from both T1–T5 and T1–T2 models of all outcomes) testing the effect of time on QQSR. To obtain an interpretable effect size for change in the QQSR over time, we calculated Cohen's *d* as the difference between two timepoints divided by their average standard deviation (Lakens 2013), based on the T1, T2, and T5 means and variances implied by the growth curve models. To evaluate longitudinal associations between social connection and self‐affiliation, we included QQSR total and Saff in a Random‐Intercept Cross‐Lagged Panel Model (RI‐CLPM) testing whether deviation from a person‐specific level on one construct predicts subsequent deviations on another construct. The RI‐CLPM captures stable between‐person differences using a latent random intercept factor, estimating the auto‐regressive and cross‐lagged associations among the latent within‐person residuals (Hamaker et al. 2015; Mulder and Hamaker 2021). To account for differences in scale, we rescaled Saff to QQSR total (1–4). To allow for average change in the variables across time, the intercepts of observed variables were estimated freely (Falkenström et al. 2023; Mulder and Hamaker 2021). We tested equality constraints on the latent residual covariance structure after the first timepoint (i.e., stationarity assumptions) to improve parsimony and model fit. Common guidelines were used to evaluate the magnitude of misfit (Schreiber 2017). No constraints were applied to auto‐regressive or cross‐lagged effects, as the measurement occasions were not equally spaced, and treatment occurred between T1 and T2. The RI‐CLPM with a freely estimated mean structure accounts for average trends, but does not separate individual trends from cross‐lagged effects. Models that do not account for individual trends may give rise to spurious cross‐lagged effects, particularly in the presence of an intervention effect (Falkenström et al. 2023). Following Falkenström et al. recommendations (2017, 2023), we tested the robustness of cross‐lagged effects by fitting a multivariate latent growth curve model with structured residuals (LCM‐SR) (Curran et al. 2014) as a detrended version of the RI‐CLPM. We provide detailed model‐building strategies in the supplements. Finally, as a sensitivity analysis, we controlled for the effect of personality disorder on the random intercepts and slopes, by regressing these growth factors on pretreatment PD (coded as 0, 1). In all models, missing data was handled using full information maximum likelihood (FIML), assuming values are conditionally missing at random (MAR) (Enders 2022), and we accounted for clustering at the therapist‐level by estimating cluster‐robust standard errors using the “cluster” option in *lavaan*.

### 2.4 Transparency and Openness

The data used for this article were part of a large data collection from 1995 to 2008 on psychotherapy outcomes (NMSPOP). Due to several reasons including organizational obstacles, quantitative analyses of the main outcomes were not published before 2020. Registration on primary outcomes and hypotheses were done in 2019, on the Open Science Framework (Nordmo 2018), followed by publications on primary outcomes (Nordmo et al. 2020, 2021a, 2021b). In the current study, we evaluate a secondary outcome. No previous publication has evaluated QQSR as a treatment outcome, nor investigated the relationships between QQSR and SASB across time. Several publications on psychotherapy process are based on the NMSPOP dataset. Two previous NMSPOP publications are related but not overlapping with the current study: Hersoug et al. (2002) investigated whether baseline QQSR predicted session 3 and session 12 working alliance in a subsample of NMSPOP (*N* = 270). It did not evaluate QQSR beyond the first time point, unlike the current study. Halvorsen and Monsen (2007) investigated change in SASB categories from pre‐ to posttreatment, and whether SASB moderated treatment response in a subsample of NMSPOP (*N* = 233). The current study does not evaluate change in SASB as a research question, but only its relationship to QQSR, using long‐term follow‐up measurements in the complete *N* = 370 sample. The dataset is not publicly available due to privacy and ethical restrictions. Pending approval that all data are made anonymous and in compliance with the general data protection regulation and other local regulations, the data may be made available on request. The code used to process and analyze the data is available at the following repository: https://osf.io/8nfxc/?view_only=db1456efa8164ea39da813ea12913225.

## 3 Results

### 3.1 Social Connection Change

See Figure 1 and Supplement S1 for mean scores. All LCMs showed acceptable to excellent fit (for the total index: *χ* ^2^(10) = 12.95, *p* = 0.226; CFI = 0.997; TLI = 0.997; RMSEA = 0.028, 90% CI = [0.000, 0.067]; SRMR = 0.033). As seen in Table 1, there was significant growth in the QQSR total index from T1 to T5, alongside the support and outside family indexes, with small effect sizes, but no significant change from T1 to T2. There was significant growth in the number of current family relations from T1 to T2 and T5, and significant decrease in the number of family of origin relations from T1 to T2 and T5. Nearly all indexes, including the QQSR total, showed significant variation in slopes; see Supplement S3 for all QQSR univariate LCM results.

**Figure 1:**
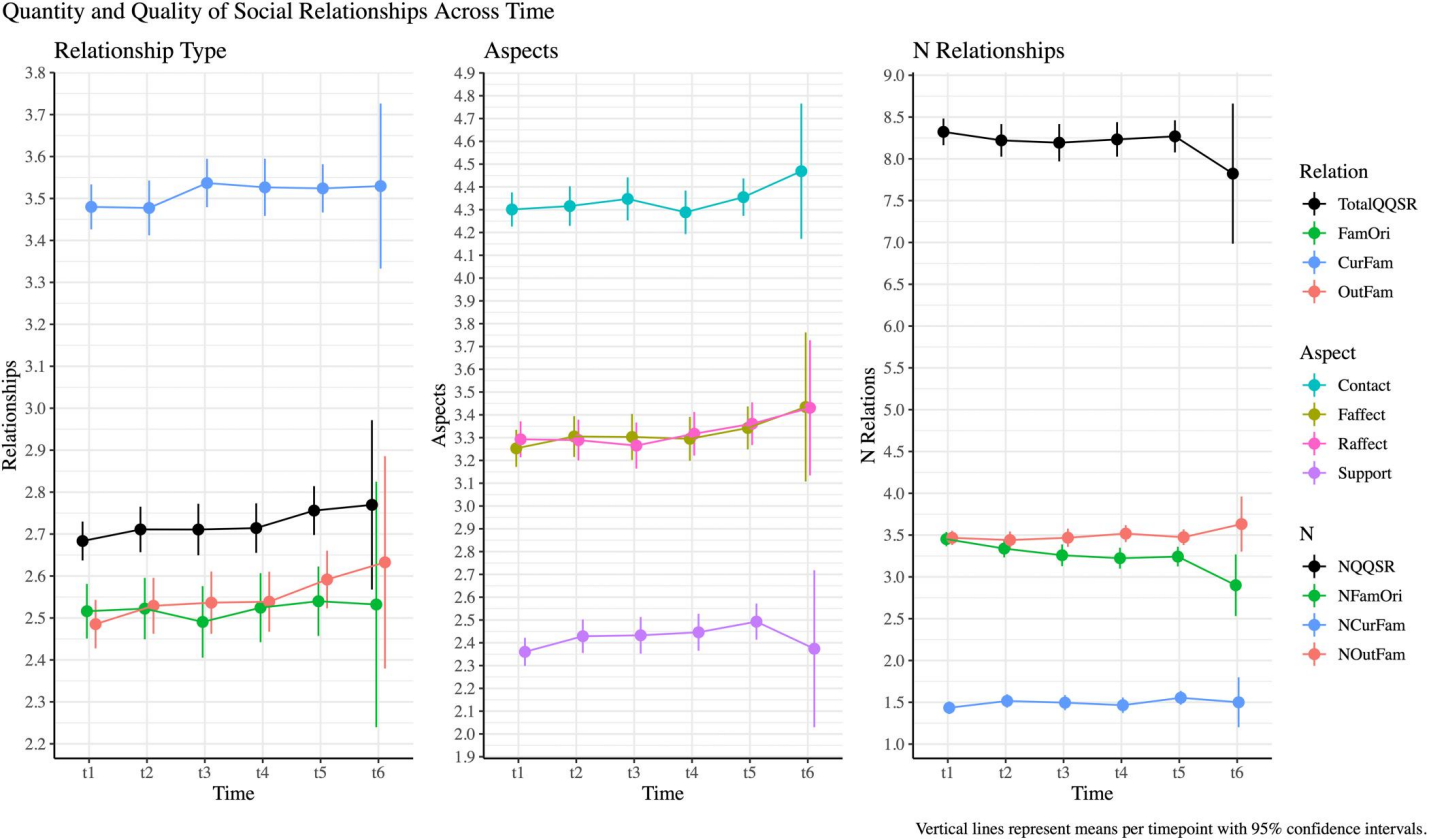
Mean quantity and quality of social relations across time. Means (points) with 95% confidence intervals (error bars). Contact range 1–6, affection indexes 1–5, total and relationship group indexes 1–4, *N* indexes range 0–10 (total), 0–4 (family of origin), 0–2 (current family), and 0–4 (outside family).

**Table 1:** QQSR growth from T1 to T5 and T1 to T2.

Index	*b* [95% CI] T5	pFDR T5	*d* T5	*b* [95% CI] T2	pFDR T2	*d* T2
TotalQQSR	0.02 [0.01, 0.03]	0.015*	0.33	0.03 [−0.02, 0.07]	0.369	0.10
Contact	0.01 [−0.01, 0.04]	0.361	0.17	0.04 [−0.03, 0.11]	0.369	0.09
Faffect	0.02 [0.00, 0.04]	0.109	0.19	0.05 [−0.04, 0.13]	0.378	0.09
Raffect	0.02 [0.00, 0.04]	0.121	0.18	0.00 [−0.07, 0.08]	0.926	0.01
Support	0.03 [0.01, 0.04]	0.014*	0.31	0.05 [−0.01, 0.12]	0.252	0.13
FamOri	0.01 [−0.01, 0.02]	0.617	0.06	0.01 [−0.04, 0.06]	0.811	0.03
CurFam	0.01 [0.00, 0.02]	0.215	0.15	0.00 [−0.06, 0.07]	0.926	0.01
OutFam	0.03 [0.01, 0.04]	0.015*	0.35	0.04 [−0.02, 0.11]	0.351	0.12
NQQSR	−0.02 [−0.06, 0.01]	0.369	−0.14	−0.06 [−0.22, 0.10]	0.617	−0.07
NFamOri	−0.05 [−0.07, −0.03]	< 0.001***	−0.50	−0.10 [−0.16, −0.04]	0.014*	−0.28
NCurFam	0.02 [0.00, 0.03]	0.047*	0.23	0.07 [0.02, 0.12]	0.042*	0.21
NOutFam	0.01 [−0.02, 0.03]	0.712	0.05	−0.02 [−0.13, 0.10]	0.811	−0.03

*Note:* Effect of time as years in random slope (T5) and difference between pretreatment and first follow‐up (T2) in random intercept latent growth curve models with 95% Confidence Interval. Cluster‐robust standard errors for therapist‐level clustering. T1 = pretreatment, T2 = post treatment, T3 = 6 months post T2, T4 = 1 year post T2, T5 = 2 and a half years post T2. *p*‐values corrected for False Discovery Rate (FDR) of all *p*‐values in the table.

Abbreviations: Contact, frequency of contact; CurFam, current family (partner and child); Faffect, freely expressed affection; FamOri, family of origin (mother, father, sibling, other relation); OutFam, outside family (friend, colleagues, neighbors); QQSR, quantity and quality of social relations; Raffect, received affection. N = number of relationships present.

* *p* < 0.05

*** *p* < 0.001.

### 3.2 Relating Social Connection and Self‐Affiliation Change

The best‐fitting parsimonious RI‐CLPM was achieved by constraining, respectively, the variances within construct and covariance between constructs to be equal at T2–T5 (*χ* ^2^(30) = 55.47, *p* = 0.003; CFI = 0.987; TLI = 0.981; RMSEA = 0.048, 90% CI = [0.027, 0.067]; SRMR = 0.052). There were three significant cross‐lagged effects: higher T2 QQSR predicted higher T3 Saff (standardized regression coefficient b* = 0.27, 95% CI = [0.13, 0.40], *p* < 0.001), higher T1 Saff predicted lower T2 QQSR (b* = −0.29 [−0.50, −0.08], *p* = 0.022), and higher T3 Saff predicted higher T4 QQSR (b* = 0.36 [0.17, 0.55], *p* < 0.001). Random intercepts were significantly correlated (*r* = 0.44 [0.34, 0.53], *p* < 0.001), as were the latent residuals of the two variables through T2–T5 (*r* = 0.23 [0.13, 0.33], *p* < 0.001). Both intercepts showed significant variance. See Supplement S4 for model building strategy and all RI‐CLPM results.

The best‐fitting parsimonious LCM‐SR was achieved using the same constraints as the final RI‐CLPM, with the log of time as the slope factor for Saff, and a linear slope of time for QQSR, as a detrended version of the RI‐CLPM. The LCM‐SR achieved equally good fit (*χ* ^2^(29) = 52.40, *p* = 0.005; CFI = 0.988; TLI = 0.982; RMSEA = 0.047, 90% CI = [0.025, 0.067]; SRMR = 0.042). The Akaike Information Criterion favored the LCM‐SR (AIC = 1814.6, BIC = 1955.5), while the Bayesian Information Criterion, which penalizes model complexity, favored the RI‐CLPM (AIC = 1815.7, BIC = 1952.6). After detrending, there were two remaining significant cross‐lagged effects: higher T2 QQSR predicted higher T3 Saff (b* = 0.32, 95% CI = [0.17, 0.48], *p* < 0.001), and higher T3 Saff predicted higher T4 QQSR (b* = 0.32 [0.11, 0.53], *p* = 0.002), as displayed in Figure 2. There were significant positive correlations between the random intercepts (*r* = 0.31 [0.12, 0.50], *p* = 0.004), and between the QQSR intercept and the Saff slope (*r* = 0.25 [0.00, 0.49], *p* = 0.042), as well as significant correlations between the latent residuals at each wave. There was significant variance in the Saff intercept and slope, and in the QQSR intercept but not slope. All growth factor means were significantly different from 0 (QQSR intercept = 2.68 [2.64, 2.73], *p* < 0.001; QQSR slope = 0.02 [0.01, 0.03], *p* = 0.002 for time in years; Saff intercept = 2.56 [2.51, 2.60], *p* < 0.001; Saff slope = 0.10 [0.09, 0.12], *p* < 0.001 for the log of time in months). In the sensitivity analysis controlling for T1 PD, the results were similar with the same inferences for cross‐lagged paths. Pretreatment PD showed a significant effect on both intercepts (QQSR *b* = −0.23 [−0.31, −0.14], *p* < 0.001; Saff *b* = −0.31 [−0.41, −0.22], *p* < 0.001) but not slopes, suggesting that patients with a PD had less social connection and a more hostile self‐relationship, but equal rates of change. See Supplement S4 for model building strategy and all LCM‐SR results.

**Figure 2:**
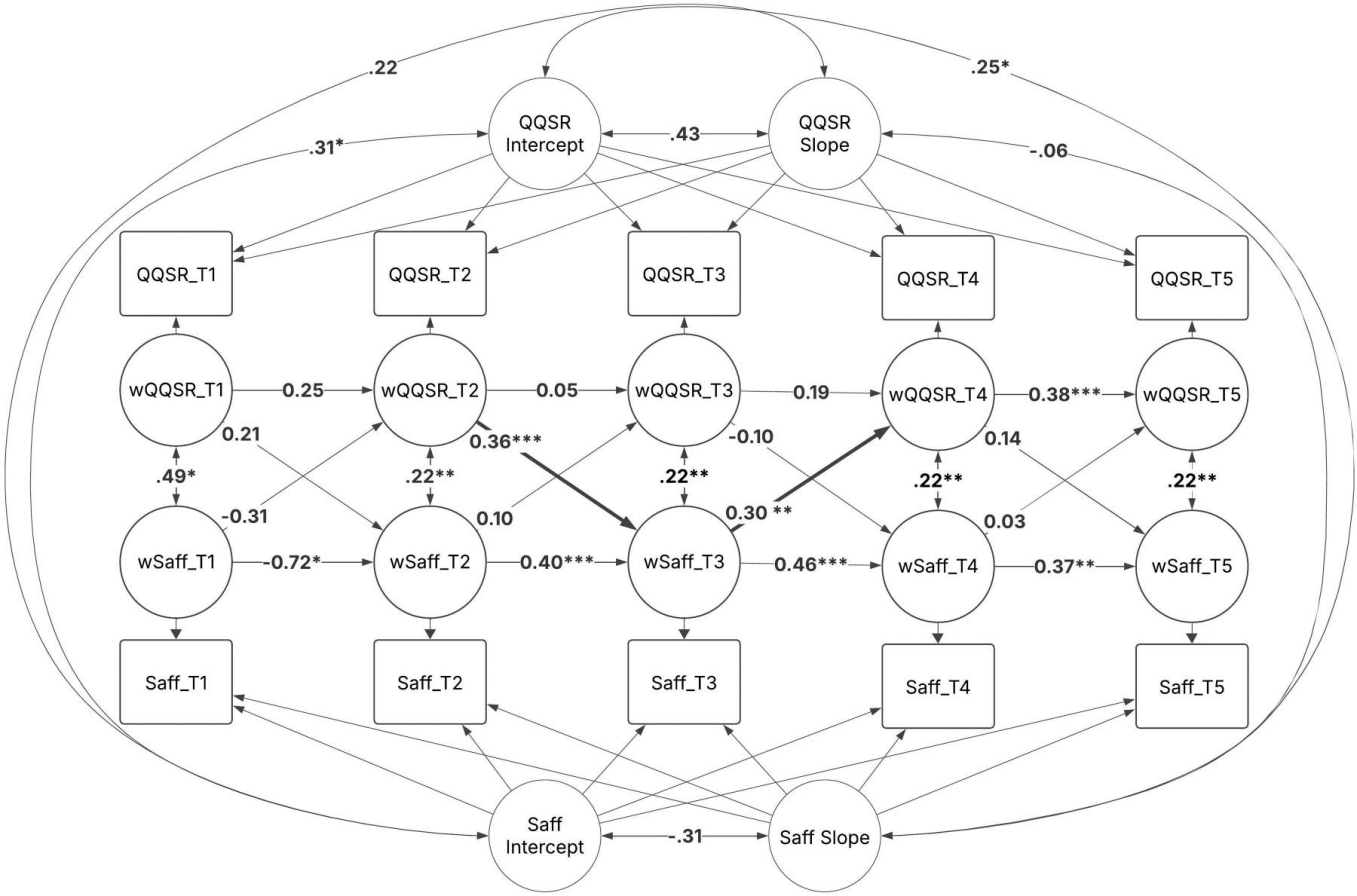
Multivariate latent curve model with structured residuals. wQQSR and wSaff = within‐person latent residuals. Significant cross‐lagged paths are highlighted. Variances of each within‐person latent variable, and their covariance at each wave, were constrained to be equal across waves T2 to T5. Covariances are correlations; regressions are unstandardized. *p < 0.05, **p < 0.01, ***p < 0.001. Not displayed in figure: intercepts, variances and factor loadings defining the latent variables; see Supplement S4 for full results.

## 4 Discussion

We found evidence of small, gradual improvements from baseline through long‐term follow‐up in global social connection, the support aspect, and non‐family relationships. We deem the significant changes in numbers of relation indexes (family of origin and current family) to correspond to common life changes (e.g., having children, losing parents) and do not interpret them further.

Contrary to our hypothesis, we did not find change in social connection from pretreatment to the first follow‐up. The open‐ended psychotherapy provided, which did not specifically target social connection, may have been ineffective at improving this outcome as measured by the QQSR. This is consistent with a prior publication demonstrating minimal change in occupational functioning in the NMSPOP sample (Nordmo et al. 2020). In contrast to our findings, meta‐analytic evidence suggests that psychological interventions can improve social connection (Zagic et al. 2022). In addition to the non‐targeted treatment, the lack of change could also be due to characteristics of the sampled population or of the measure. For example, Cregg and Cheavens (2023) showed that a less intensive intervention targeting social connection induced significant change on the more recent revised Social Connectedness Scale (SCS; Lee et al. 2001). However, the sample consisted of undergraduate students with elevated symptoms of anxiety or depression. A recent naturalistic study more similar to NMSPOP showed significant improvement in social support satisfaction measured by the Brief Inventory of Social Support and Integration scale (BISSI; Lindfors et al. 2014) during open‐ended psychodynamic therapy. However, this study excluded patients with PD, and around 80% of the sample were working or studying. In contrast to these studies, roughly half of the NMSPOP sample presented with PD at baseline and about half of the sample were working or studying.

The NMSPOP sample has shown significant change at first follow‐up in other outcomes, including interpersonal problems, as well as symptoms, diagnoses and personality pathology (Nordmo et al. 2020). The QQSR could be measuring more enduring or trait‐like aspects of specific dyadic relationships. We may speculate that scales measuring a patient's perception of aspects of their social connection *in general* may be more sensitive, such as interpersonal problem inventories (Horowitz et al. 1988), or scales of social connectedness (Lee et al. 2001), loneliness (Hickin et al. 2021), or support satisfaction (Lindfors et al. 2014). Finally, the lack of change could be due to psychometric limitations; the QQSR items might not be sufficiently sensitive to more subtle changes in the specified relationships.

Patient heterogeneity might also have contributed to the modest group‐level change rates. Substantial variance in slopes suggests future research might investigate why particular patients experience growth in social connection after psychotherapy. For example, in the LCM‐SR, the variance in slopes was no longer significant, suggesting that the self‐affiliation growth factors and within‐person residuals might account for some of the between‐person variation in change in social connection. Future research might attempt to disentangle the heterogeneity between studies on social connection growth during psychotherapy at the patient‐, treatment‐ and therapist‐ levels.

The findings suggest a slow, posttreatment growth process in which patients improve in their capacity to elicit support. We speculate that patients benefit from healing experiences in which they express needs and receive support from a responsive, affirming, and empathic therapist (Norcross and Lambert 2019), which may generalize to extra‐therapy relationships over time. Having needs validated and met by the therapist and others may over time make it easier to seek support from others, in turn contributing to a kinder relationship to oneself. The results further suggest growth in patients' connection to colleagues, people in their community, and friends. While such non‐romantic and non‐familial relationships have often been overlooked in outcome research, interaction with such “weak ties” is associated with well‐being (Sandstrom and Dunn 2014) and friendships have unique effects on health (Vieth et al. 2025).

Thus, despite our clinical impression and converging meta‐analytic evidence (Kidd et al. 2022) that distress regarding one's family of origin is common for patients in psychotherapy, our findings suggest these relationships are more crystallized and less amenable to change, at least in the aspects measured by the QQSR. We further propose that the observed stability in the current family index may stem from ceiling effects in responses, as noted in the Methods section. Thus, the QQSR is likely not sensitive as a measure of the quality of partner and child relationships.

Patients with more positive social relationships also had a more friendly relationship to themselves. Over time within patients, they show bi‐directional lagged associations when controlling for auto‐regressive effects. Because cross‐lagged paths in RI‐CLPMs may be spurious in the presence of trends, we interpret only those that remained significant after detrending with the LCM‐SR (Falkenström et al. 2023). Consistent with our hypothesis, we found significant positive reciprocal cross‐lagged effects, but only between three of the five timepoints. Contrary to our hypothesis, improved self‐affiliation on the first follow‐up after treatment did not predict improved social connection. Instead, our findings suggest that improved social connection on the first follow‐up predicts the subsequent development of a more friendly relationship to oneself. Then, developing a more friendly self‐relationship in the next follow‐up seems to facilitate subsequent improved social connection. In addition, a significant cross‐construct intercept–slope correlation suggested that patients starting therapy with low social connection may experience less improvement in their self‐relationship. This is consistent with previous studies showing in particular that pretreatment social support predicts psychotherapy process and outcomes (Lindfors et al. 2014; Roehrle and Strouse 2008; Zimmermann et al. 2021). Patients with more social support may have more resources to make use of therapy, and positive extratherapeutic relationships might in themselves contribute to recovery from a negative self‐relationship (Roehrle and Strouse 2008).

A clinical implication of our findings is that therapists might encourage patients' development of meaningful extratherapeutic relationships to potentiate therapeutic work on patients' self‐concept. Therapists might screen the quality of a client's social relationships at the outset of therapy and adjust their interventions accordingly. For example, during treatment, therapists might help patients identify where they could seek social relationships that meet their needs. Similarly, therapists might examine whether and how the way patients treat themselves inhibits them from developing more satisfying extratherapeutic relationships that can sustain them after therapy ends.

The findings suggest a virtuous cycle of inter‐ and intrapersonal relatedness. Further research might explore what kinds of interventions and processes are conducive to this process, and proximal session‐to‐session associations between relationships to self, extratherapeutic relationships to others, and the relationship to the therapist (Zimmermann et al. 2021). It remains an open question how self‐ and other‐relationships are associated in treatments specifically targeting these relationships as mechanisms of change (e.g., compassion‐focused therapies, interpersonal reconstructive therapy). Future research might also investigate the clinical utility of the QQSR, e.g., how the QQSR can contribute to case formulation and therapist‐client communication at the start of treatment.

## 5 Limitations

This study has several methodological limitations that should be mentioned. The naturalistic dataset is unavoidably characterized by sources of noise such as different treatment lengths, auxiliary treatments, numbers and timings of measurements. A major limitation on generalizability is the exclusion of patients with psychosis and substance use disorders, particularly given the importance of social connection to recovery from these disorders. Another major limitation on generalizability is the considerable age of the dataset, representing a Norwegian patient and therapist sample from 1995 to 2008. It is possible the observed results would be different if the data had been collected more recently. We identified ceiling effects in current family items, suggesting that we did not have a sensitive measure for detecting change in these relationships. The study was not pre‐registered and serves foremost to generate hypotheses for future studies. Lacking a control group, the observed effects cannot necessarily be attributed to psychotherapy. Neither therapists nor patients were randomized to participate, meaning selection effects can influence generalizability. Missing data handling methods rely on MAR assumptions which may not hold. To reduce model complexity, we did not account for site‐level nesting. The study is limited by the competing explanations for the lack of social connection change from pre‐ to posttreatment. Finally, the QQSR has limitations compared to other measures of social connection. The QQSR can provide relatively granular information about a patient's social connection by assessing a variety of aspects across a broad range of specific relationships. However, the specific relationship categories may omit important sources of social connection sampled by other measures, and might be less sensitive compared to scales focusing on assessing the quality of a particular relationship (e.g., romantic relationship satisfaction) or scales measuring the patient's perception of their social connection (e.g., loneliness scales). Currently, several other social connection measures have more psychometric evidence (Plackett et al. 2024).

## 6 Conclusion

We draw the following conclusions: (1) on average, social connection, in particular social support and non‐family relationships, improved gradually from pretreatment across follow‐up in open‐ended psychotherapy, with substantial variation; (2) improved social connection after treatment might cause improvements in self‐affiliation during follow‐up, which in turn might cause improved social connection; (3) the QQSR might measure relatively stable aspects of a person's network and relevant between‐person differences. These pretreatment differences may be relevant to therapeutic self‐relationship growth; (4) clinicians might screen for social and self‐relationships before and throughout therapy, attend to how they influence each other for a particular patient, and consider them both as treatment outcomes and resources.

## Ethics Statement

The Regional Committee for Medical Research Ethics in Eastern Norway approved the study (approval number S‐95010).

## Conflicts of Interest

The authors report there are no competing interests to declare.

## Supporting information


Supporting File 1

Supporting File 2

Supporting File 3

Supporting File 4

## Data Availability

The dataset is not publicly available due to privacy and ethical restrictions. Pending approval that all data are made anonymous and in compliance with the general data protection regulation and other local regulations, the data may be made available on request.
