# Supplementary material for: Connected to Others and Kind to Self: Social Connection and Self‐Affiliation Change in Open‐Ended Psychotherapy
Source: J Clin Psychol. 2026 Sep 4;82(12):2060–9. doi: 10.1002/jclp.70220 (PMC13733646; doi:10.1002/jclp.70220)
Supplement: Supplementary file 1 [file JCLP-82-2060-s001.docx]

### Supplement 02

### Evaluating the Convergent Validity of the QQSR

### Variables for Validating the QQSR

*The Structural Analysis of Social Behaviour - Introject Surface (SASB-I)* contains 36 items describing how a person treats themselves. It consists of eight subscales along a circumplex based on the dimensions self-autonomy and self-affiliation (Benjamin, 1996). Patients were instructed to rate “yourself as you usually are”. Self-affiliation and -autonomy were calculated as weighted sums of the subscales (Pincus et al., 1998). Higher scores indicate, respectively, more loving or autonomous self-treatment, and lower scores more attacking or controlling self-directed behaviour. *The Symptom Checklist 90 Revised (SCL-90-R)* is a self-report measure of symptoms during the last week (Derogatis & Unger, 2010). The mean Global Severity Index (GSI) was used. *The Inventory of Interpersonal Problems (IIP-64)* is a self-report measure of interpersonal problems (Horowitz et al., 1988), consisting of eight subscales along the two dimensions of autonomy and affiliation. The mean score was used. The clinician-rated *Global Assessment of Functioning (GAF)* was used as a measure of overall patient functioning (Endicott et al., 1976). *Personality disorders (PD)* were assessed according to the DSM-IV (American Psychiatric Association, 1994) with SCID II semi-structured interview (Lobbestael et al., 2011), as described in a previous study (Nordmo et al., 2020). We used both the presence or absence of PD and the number of above threshold PD criteria. *Dynamic Scales (DS)* comprise clinician-rated patient functioning in five domains, based on a semi-structured interview (Høglend et al., 2000). *The Affect Consciousness Interview (ACI)* is a semi-structured interview where clinicians rate the patient’s awareness, tolerance, nonverbal and verbal expression for nine discrete affects: Interest, Joy, Tenderness/Care, Fear, Anger, Shame, Sadness, Envy, Guilt (Solbakken et al., 2011). Each of the four aspects is rated for each affect, yielding 39 indicators that can be summarized by a global mean score (Global Affect Consciousness), and mean aspect and affect scores. A subset of patients (N = 153) were interviewed with the ACI at T1. *The Parental Bonding Instrument (PBI)* is a self-report measure of the perceived parenting style of one’s parents during the first 16 years of life, on the dimensions of care and overprotection (Parker et al., 1979). The short-form Patient-rated *Working Alliance Inventory (WAI-P)* is a 12-item measure of the relationship between the patient and therapist (Tracey & Kokotovic, 1989). The mean score was used. The WAI-P from the third psychotherapy session was used. Other measures were measured concurrently with QQSR at baseline (T1). Bivariate correlations were then used to examine evidence of convergent validity. Correlations with t-tests were performed with *Hmisc* (Harrell, 2025).

| **Tables** | | | | | | | | | |
| --- | --- | --- | --- | --- | --- | --- | --- | --- | --- |
| *Baseline Correlations between QQSR and Psychiatric Symptoms, Personality Pathology, Functioning and Early Alliance* | | | | | | | | | |
| Variable | GSI | PDsum | GAF | dynFri | dynRom | dynTol | dynIns | dynAda | WAIP3 |
| TotalQQSR | -0.18*** | -0.31*** | 0.27*** | 0.45*** | 0.22*** | 0.28*** | 0.22*** | 0.33*** | 0.21*** |
| Contact | -0.05 | -0.18*** | 0.22*** | 0.36*** | 0.17** | 0.21*** | 0.17** | 0.24*** | 0.24*** |
| Faffect | -0.18*** | -0.28*** | 0.25*** | 0.39*** | 0.19*** | 0.24*** | 0.22*** | 0.30*** | 0.10 |
| Raffect | -0.16** | -0.25*** | 0.22*** | 0.32*** | 0.12* | 0.20*** | 0.13* | 0.24*** | 0.12* |
| Support | -0.14** | -0.25*** | 0.18*** | 0.36*** | 0.22*** | 0.24*** | 0.19*** | 0.27*** | 0.21*** |
| FamOri | -0.17*** | -0.26*** | 0.20*** | 0.34*** | 0.11* | 0.23*** | 0.14** | 0.25*** | 0.21*** |
| CurFam | -0.14* | -0.15** | 0.15** | 0.20*** | 0.23*** | 0.16** | 0.11* | 0.19*** | 0.11 |
| OutFam | -0.14** | -0.22*** | 0.24*** | 0.38*** | 0.14** | 0.23*** | 0.21*** | 0.28*** | 0.10 |
| NQQSR | -0.13* | -0.07 | 0.16** | 0.22*** | 0.33*** | 0.23*** | 0.15* | 0.18** | 0.03 |
| NFamOri | -0.03 | 0.09 | -0.03 | -0.04 | -0.05 | 0.00 | -0.02 | -0.06 | -0.05 |
| NCurFam | -0.04 | -0.08 | 0.10 | 0.20*** | 0.39*** | 0.14** | 0.13* | 0.16** | 0.03 |
| NOutFam | -0.15** | -0.15* | 0.24*** | 0.23*** | 0.20*** | 0.21*** | 0.13* | 0.23*** | 0.07 |
| *Note:* Pearson correlations for continuous indexes, and Spearman correlations for the N indexes. All scores are from pretreatment baseline (T1) except the WAI. QQSR: Quantity and Quality of Social Relationships, Contact: frequency of contact, Faffect: freely expressed affection, Raffect: received affection, FamOri: family of origin (mother, father, sibling, other relation), CurFam: current family (partner and child), OutFam: outside family (female friend, male friend, colleagues, neighbours), N: number of relations present in the patient's life (total 0 to 9). GSI: SCL-90 Global Severity Index, PDsum: number of above threshold DSM-IV SCID-II Personality Disorder criteria, GAF: Global Assessment of Functioning, dynFri to dynAda: Dynamic Scales: Quality of Friendship/Family relations, Romantic/sexual relationships, Tolerance for affects, Insight and Problem-solving/Adaptive capacity scales. WAIP3: patient Working Alliance Inventory at session 3. * = p<.05, ** = p<.01, *** = p <.001. | | | | | | | | | |

| *Correlations between QQSR and Interpersonal Problems at T1* | | | | | | | | | | | |
| --- | --- | --- | --- | --- | --- | --- | --- | --- | --- | --- | --- |
| Variable | PA | BC | DE | FG | HI | JK | LM | NO | OM | IIPAGE | IIPCOM |
| TotalQQSR | -0.16** | -0.27*** | -0.46*** | -0.45*** | -0.29*** | -0.14** | -0.10 | -0.01 | -0.36*** | 0.19*** | 0.35*** |
| Contact | -0.07 | -0.18*** | -0.33*** | -0.28*** | -0.20*** | -0.05 | -0.02 | -0.01 | -0.22*** | 0.12* | 0.27*** |
| Faffect | -0.20*** | -0.29*** | -0.47*** | -0.44*** | -0.26*** | -0.15** | -0.12* | -0.04 | -0.37*** | 0.16** | 0.34*** |
| Raffect | -0.14** | -0.21*** | -0.40*** | -0.40*** | -0.26*** | -0.13* | -0.11* | -0.05 | -0.33*** | 0.17*** | 0.28*** |
| Support | -0.11* | -0.19*** | -0.29*** | -0.32*** | -0.19*** | -0.10 | -0.07 | 0.06 | -0.23*** | 0.15** | 0.25*** |
| FamOri | -0.19*** | -0.21*** | -0.36*** | -0.34*** | -0.25*** | -0.15** | -0.13* | -0.07 | -0.32*** | 0.14** | 0.22*** |
| CurFam | -0.15** | -0.14* | -0.23*** | -0.18** | -0.10 | -0.11 | -0.11* | -0.03 | -0.19*** | 0.04 | 0.11* |
| OutFam | -0.06 | -0.21*** | -0.35*** | -0.40*** | -0.21*** | -0.06 | -0.03 | 0.06 | -0.25*** | 0.18*** | 0.34*** |
| NQQSR | 0.14* | -0.02 | -0.14* | -0.12* | -0.09 | -0.09 | -0.03 | 0.05 | -0.06 | 0.15** | 0.06 |
| NFamOri | 0.02 | 0.05 | 0.04 | 0.06 | 0.03 | -0.03 | -0.03 | 0.03 | 0.04 | 0.02 | -0.05 |
| NCurFam | 0.02 | -0.09 | -0.17** | -0.14** | -0.11* | -0.06 | 0.01 | -0.02 | -0.10 | 0.09 | 0.10 |
| NOutFam | 0.09 | -0.09 | -0.15** | -0.16** | -0.11* | -0.06 | -0.01 | 0.05 | -0.09 | 0.14* | 0.11* |
| *Note:* Pearson correlations for continuous indexes and Spearman correlations for the N indexes. QQSR: Quantity and Quality of Social Relationships, Contact: frequency of contact, Faffect: freely expressed affection, Raffect: received affection, FamOri: family of origin (mother, father, sibling, other relation), CurFam: current family (partner and child), OutFam: outside family (female friend, male friend, colleagues, neighbours), N: number of relations present in the patient's life (total 0 to 9). Interpersonal problems were measured with the Inventory of Interpersonal Problems (IIP-64). OM: global elevation of interpersonal distress. IIP-64 subscales: pa: domineering, bc: vindictive, de: cold, fg: socially inhibited, hi: nonassertive, jk: overly accommodating, lm: self-sacrificing, no: intrusive, IIPAGE: agency dimension, IIPCOM: communion dimension. * = p<.05, ** = p<.01, *** = p <.001. | | | | | | | | | | | |

| *Correlations between QQSR, Self-Relatedness and Affect Integration at T1* | | | | | | | | | | | | | | |
| --- | --- | --- | --- | --- | --- | --- | --- | --- | --- | --- | --- | --- | --- | --- |
| Variable | AC | In | Jo | Ca | Fe | An | Sh | Sa | En | Gu | Aw | Tol | Em | Ve |
| TotalQQSR | 0.27*** | 0.08 | 0.25** | 0.28*** | 0.21** | 0.18* | 0.14 | 0.24** | 0.02 | 0.17* | 0.04 | 0.32*** | 0.25** | 0.28*** |
| Contact | 0.18* | 0.10 | 0.19* | 0.20* | 0.18* | 0.14 | 0.04 | 0.14 | -0.01 | 0.10 | 0.03 | 0.19* | 0.16* | 0.22** |
| Faffect | 0.21** | 0.07 | 0.23** | 0.24** | 0.18* | 0.12 | 0.11 | 0.14 | 0.00 | 0.16* | 0.02 | 0.31*** | 0.17* | 0.21** |
| Raffect | 0.19* | 0.02 | 0.16* | 0.22** | 0.10 | 0.12 | 0.10 | 0.19* | 0.03 | 0.14 | -0.00 | 0.28*** | 0.16* | 0.19* |
| Support | 0.27*** | 0.07 | 0.22** | 0.25** | 0.21** | 0.18* | 0.17* | 0.29*** | 0.03 | 0.14 | 0.10 | 0.22** | 0.29*** | 0.29*** |
| FamOri | 0.19* | 0.09 | 0.15 | 0.19* | 0.16* | 0.18* | 0.03 | 0.22** | -0.03 | 0.09 | 0.05 | 0.25** | 0.15 | 0.18* |
| CurFam | 0.22** | 0.10 | 0.25** | 0.20* | 0.16 | 0.07 | 0.17 | 0.20* | 0.07 | 0.06 | 0.15 | 0.20* | 0.22** | 0.18* |
| OutFam | 0.19* | 0.02 | 0.20* | 0.21** | 0.14 | 0.11 | 0.16* | 0.12 | 0.04 | 0.14 | 0.03 | 0.22** | 0.18* | 0.21* |
| NQQSR | 0.15 | 0.03 | 0.07 | 0.06 | 0.16 | 0.11 | 0.12 | 0.06 | 0.19* | 0.10 | 0.14 | 0.10 | 0.11 | 0.17 |
| NFamOri | -0.06 | 0.01 | -0.06 | -0.11 | -0.06 | -0.03 | 0.02 | -0.02 | 0.04 | -0.09 | 0.04 | -0.13 | -0.04 | -0.06 |
| NCurFam | 0.15 | 0.03 | 0.13 | 0.04 | 0.14 | 0.09 | 0.05 | 0.12 | 0.12 | 0.22** | 0.03 | 0.15 | 0.11 | 0.20* |
| NOutFam | 0.10 | -0.01 | -0.02 | 0.15 | 0.10 | 0.02 | 0.12 | 0.03 | 0.06 | 0.09 | 0.13 | 0.07 | 0.06 | 0.12 |
| *Note:* Pearson correlations for continuous indexes and Spearman correlations for the N indexes. QQSR: Quantity and Quality of Social Relationships, Contact: frequency of contact, Faffect: freely expressed affection, Raffect: received affection, FamOri: family of origin (mother, father, sibling, other relation), CurFam: current family (partner and child), OutFam: outside family (female friend, male friend, colleagues, neighbours), N: number of relations present in the patient's life (total 0 to 9). Affect integration was measured with the Affect Consciousness Interview (ACI). AC: global affect consciousness score. ACI emotion subscales: In: interest, Jo: joy, Ca: tenderness/care, Fe: fear, An: anger, Sh: shame, Sa: sadness, En: envy, Gu: guilt. ACI aspects: Aw: awareness, Tol: tolerance, Em: nonverbal/emotional expression, Ve: verbal/conceptual expression. * = p<.05, ** = p<.01, *** = p <.001. | | | | | | | | | | | | | | |

| *Correlations between QQSR and Self-Relatedness at T1* | | | | | | | | | | |
| --- | --- | --- | --- | --- | --- | --- | --- | --- | --- | --- |
| Variable | Saff | Saut | Emancipate | Affirm | Love | Protect | Control | Blame | Attack | Ignore |
| TotalQQSR | 0.34*** | 0.19*** | 0.06 | 0.34*** | 0.34*** | 0.15** | -0.16** | -0.24*** | -0.20*** | -0.19*** |
| Contact | 0.12* | 0.05 | 0.01 | 0.11* | 0.12* | 0.03 | -0.05 | -0.08 | -0.07 | -0.10* |
| Faffect | 0.31*** | 0.20*** | 0.05 | 0.31*** | 0.31*** | 0.15** | -0.20*** | -0.22*** | -0.18*** | -0.17** |
| Raffect | 0.31*** | 0.24*** | 0.09 | 0.31*** | 0.30*** | 0.13* | -0.21*** | -0.25*** | -0.18*** | -0.17*** |
| Support | 0.31*** | 0.11* | 0.03 | 0.32*** | 0.32*** | 0.15** | -0.04 | -0.19*** | -0.19*** | -0.16** |
| FamOri | 0.29*** | 0.17** | 0.09 | 0.27*** | 0.32*** | 0.15** | -0.12* | -0.20*** | -0.16** | -0.13* |
| CurFam | 0.24*** | 0.13* | -0.04 | 0.19*** | 0.16** | 0.12* | -0.14* | -0.23*** | -0.21*** | -0.10 |
| OutFam | 0.22*** | 0.13* | 0.04 | 0.26*** | 0.23*** | 0.11* | -0.11* | -0.14** | -0.08 | -0.13* |
| NQQSR | 0.04 | -0.02 | -0.05 | 0.03 | 0.02 | 0.05 | -0.04 | -0.03 | 0.01 | -0.05 |
| NFamOri | -0.10 | -0.05 | -0.03 | -0.10 | -0.09 | 0.03 | -0.03 | 0.08 | 0.09 | 0.08 |
| NCurFam | 0.05 | -0.02 | -0.14** | 0.04 | -0.02 | -0.07 | -0.04 | -0.06 | -0.07 | -0.13* |
| NOutFam | 0.11 | 0.01 | 0.03 | 0.13* | 0.12* | 0.05 | 0.02 | -0.08 | 0.00 | -0.06 |
| *Note:* Pearson correlations for continuous indexes and Spearman correlations for the N indexes. QQSR: Quantity and Quality of Social Relationships, Contact: frequency of contact, Faffect: freely expressed affection, Raffect: received affection, FamOri: family of origin (mother, father, sibling, other relation), CurFam: current family (partner and child), OutFam: outside family (female friend, male friend, colleagues, neighbours), N: number of relations present in the patient's life (total 0 to 9). SASB introject scales: Saff: self-affiliation, Saut: self-autonomy, Emancipate to Ignore: octant subscales from self-emancipate to self-ignore. * = p<.05, ** = p<.01, *** = p <.001. | | | | | | | | | | |

| *Correlations between QQSR and Parental Bonding at T1* | | | | |
| --- | --- | --- | --- | --- |
| Variable | PC | PO | MC | MO |
| TotalQQSR | 0.26*** | -0.11* | 0.42*** | -0.18*** |
| Contact | 0.18*** | -0.04 | 0.29*** | -0.12* |
| Faffect | 0.23*** | -0.08 | 0.38*** | -0.17*** |
| Raffect | 0.23*** | -0.11* | 0.36*** | -0.17** |
| Support | 0.18*** | -0.08 | 0.29*** | -0.10* |
| FamOri | 0.36*** | -0.17*** | 0.51*** | -0.22*** |
| CurFam | 0.07 | -0.07 | 0.24*** | -0.17** |
| OutFam | 0.10 | -0.03 | 0.19*** | -0.05 |
| NQQSR | 0.02 | -0.03 | -0.08 | 0.02 |
| NFamOri | 0.03 | -0.04 | -0.03 | -0.01 |
| NCurFam | -0.07 | 0.05 | -0.07 | -0.00 |
| NOutFam | 0.11 | -0.05 | 0.03 | -0.01 |
| *Note:* Pearson correlations for continuous indexes and Spearman correlations for the N indexes. QQSR: Quantity and Quality of Social Relationships, Contact: frequency of contact, Faffect: freely expressed affection, Raffect: received affection, FamOri: family of origin (mother, father, sibling, other relation), CurFam: current family (partner and child), OutFam: outside family (female friend, male friend, colleagues, neighbours), N: number of relations present in the patient's life (total 0 to 9). PBI scales: PC: paternal care, PO: paternal overprotection, MC: maternal care, MO: maternal overprotection. * = p<.05, ** = p<.01, *** = p <.001. | | | | |

**References**

American Psychiatric Association. (1994). *Diagnostic and statistical manual of mental disorders: DSM IV. 4th ed.* American psychiatric association Washington, DC.

Benjamin, L. S. (1996). A Clinician-Friendly Version of the Interpersonal Circumplex: Structural Analysis of Social Behavior (SASB). *Journal of Personality Assessment*, *66*(2), 248–266. https://doi.org/10.1207/s15327752jpa6602_5

Derogatis, L. R., & Unger, R. (2010). Symptom checklist-90-revised. *The Corsini Encyclopedia of Psychology*, 1–2.

Endicott, J., Spitzer, R. L., Fleiss, J. L., & Cohen, J. (1976). The Global Assessment Scale: A procedure for measuring overall severity of psychiatric disturbance. *Archives of General Psychiatry*, *33*(6), 766–771.

Harrell, F. E. (2025). *Hmisc: Harrell Miscellaneous*. https://github.com/harrelfe/hmisc

Høglend, P., Bøgwald, K.-P., Amlo, S., Heyerdahl, O., Sørbye, Ø., Marble, A., Sjaastad, M. C., & Bentsen, H. (2000). Assessment of Change in Dynamic Psychotherapy. *The Journal of Psychotherapy Practice and Research*, *9*(4), 190–199.

Horowitz, L. M., Rosenberg, S. E., Baer, B. A., Ureño, G., & Villaseñor, V. S. (1988). Inventory of interpersonal problems: Psychometric properties and clinical applications. *Journal of Consulting and Clinical Psychology*, *56*(6), 885. https://doi.org/d64qkj

Lobbestael, J., Leurgans, M., & Arntz, A. (2011). Inter-rater reliability of the Structured Clinical Interview for DSM-IV Axis I Disorders (SCID I) and Axis II Disorders (SCID II). *Clinical Psychology & Psychotherapy*, *18*(1), 75–79. https://doi.org/10.1002/cpp.693

Nordmo, M., Sønderland, N. M., Havik, O. E., Eilertsen, D.-E., Monsen, J. T., & Solbakken, O. A. (2020). Effectiveness of open-ended psychotherapy under clinically representative conditions. *Frontiers in Psychiatry*, *11*, 384. https://doi.org/10/gg4gqc

Parker, G., Tupling, H., & Brown, L. B. (1979). A parental bonding instrument. *British Journal of Medical Psychology*. https://doi.org/10/cznfv5

Pincus, A. L., Newes, S. L., Dickinson, K. A., & Ruiz, M. A. (1998). A Comparison of Three Indexes to Assess the Dimensions of Structural Analysis of Social Behavior. *Journal of Personality Assessment*, *70*(1), 145–170. https://doi.org/10/frmjvg

Solbakken, O. A., Hansen, R. S., Havik, O. E., & Monsen, J. T. (2011). Assessment of affect integration: Validation of the affect consciousness construct. *Journal of Personality Assessment*, *93*(3), 257–265.

Tracey, T., & Kokotovic, A. (1989). Factor Structure of the Working Alliance Inventory. *Psychological Assessment: A Journal of Consulting and Clinical Psychology*, *1*, 207–210. https://doi.org/10.1037/1040-3590.1.3.207
