# Supplementary material for: Connected to Others and Kind to Self: Social Connection and Self‐Affiliation Change in Open‐Ended Psychotherapy
Source: J Clin Psychol. 2026 Sep 4;82(12):2060–9. doi: 10.1002/jclp.70220 (PMC13733646; doi:10.1002/jclp.70220)
Supplement: Supplementary file 2 [file JCLP-82-2060-s002.docx]

### Supplement 04

### Random Intercept Cross-Lagged Panel Model and Multivariate Latent Curve Model with Structured Residuals

This document contains model-building strategy and fit indices, arriving at the final models reported in the manuscript, followed by results tables for those models.

# Model Building Strategy

All models were clustered by therapist in the R package *lavaan*. In all models, the intercept was defined by factor loadings of 1 on all indicators, and latent residuals at each timepoint defined by a factor loading of 1 on the corresponding observed variable at that timepoint. Due to the differences in timelags, and the centrality of cross-lagged paths to our research question, we did not attempt constraints on cross-lagged paths. We rescaled Saff to share the metric of the QQSR (1 to 4).

## Random Intercept Cross-Lagged Panel Model (RI-CLPM)

To define the RI-CLPMs, the mean structure of latent variables was fixed to 0, while the mean structure of the observed variables was estimated freely, to account for the average change over time across the whole sample. The random intercepts of the two outcomes were allowed to covary. The variance of random intercepts were estimated freely. The variance of observed variables were fixed to 0, while the variance of latent residuals were estimated freely, or constrained to be equal as in some models below. We fitted the following models:

1) **Unconstrained RI-CLPM:** Auto-regressive and cross-lagged paths, latent residuals variance at each timepoint, and residual covariances at each timepoint were freely estimated. This model achieved acceptable fit (χ²(21) = 53.48, p < .001; CFI = 0.984; TLI = 0.965; RMSEA = 0.065, 90% CI = [0.043, 0.086]; SRMR = 0.050).
2) **Constrained variances:** we attempted a more parsimonious model by constraining the within-person latent residual for each outcome to be equal at each timepoint after T1, which we allowed to be different due to its unique placement before treatment. This model achieved acceptable fit (χ²(27) = 54.72, p = 0.001; CFI = 0.986; TLI = 0.977; RMSEA = 0.053, 90% CI = [0.032, 0.073]; SRMR = 0.051). Fit was not significantly different from model 1 (Δχ²(3) = 0.87, p = 0.834).
3) **Constrained covariances:** we attempted a more parsimonious model by constraining time-specific residual covariance to be equal after T1. This model also achieved acceptable fit (χ²(24) = 54.44, p < .001; CFI = 0.985; TLI = 0.971; RMSEA = 0.059, 90% CI = [0.038, 0.079]; SRMR = 0.051). Fit was not significantly different from model 1 (Δχ²(6) = 1.08, p = 0.982).
4) **Constrained variances and covariances:** we then combined the constraints of models 3 and 4. This model achieved excellent fit (χ²(30) = 55.47, p = 0.003; CFI = 0.987; TLI = 0.981; RMSEA = 0.048, 90% CI = [0.027, 0.067]; SRMR = 0.052). Fit was not significantly different from model 1 (Δχ²(9) = 1.74, p = 0.995), and so we retained this more parsimonious model with optimal fit.

Therefore, the best-fitting parsimonious RI-CLPM was achieved by constraining, respectively, the variances within construct and covariance between constructs to be equal at T2–T5.

## Univariate Latent Curve Model with Structured Residuals (uLCM-SR)

We first determined the optimal functional form of time for the two outcomes. To define LCM-SRs, the mean structure of observed variables and latent residuals was fixed to 0, while the mean structure of latent growth factors was estimated freely. The variance of observed variables were fixed to 0, while the variance of latent residuals were estimated freely or constrained as in some models below. The variances and covariances of growth factors were estimated freely or constrained in some models as seen below. Factor loadings from growth factors were fixed to test competing models of the effect of time.

### Self-affiliation

To model self-affiliation (Saff) estimated the following models, including latent residuals in all models:

1) **Empty model:** a latent random intercept model. This model showed poor fit (χ²(13) = 291.23, p < .001; CFI = 0.711; TLI = 0.778; RMSEA = 0.241, 90% CI = [0.217, 0.265]; SRMR = 0.376).
2) **Linear model:** a model with two covarying growth factors, defining the slope factor loadings as time in years, and allowing the growth factors to covary. This model resulted in negative latent variable variances.
3) Adding a residual autocorrelation to Model 2, between the timepoints with the shortest intervals: T2 to T3, and T3 to T4 (both had 6-month intervals), and constraining it to be equal. This removed negative latent variable variances but showed poor fit (χ²(9) = 163.83, p < .001; CFI = 0.839; TLI = 0.822; RMSEA = 0.216, 90% CI = [0.188, 0.245]; SRMR = 0.151).
4) **Quadratic model:** adding a fixed quadratic growth factor to Model 2, defined by factor loadings that were the quadratic of time in years, constraining the variance and covariances of the quadratic factor to 0 (otherwise it resulted in warnings about a non-positive definite covariance matrix). The model showed poor fit (χ²(9) = 103.23, p < .001; CFI = 0.902; TLI = 0.891; RMSEA = 0.168, 90% CI = [0.140, 0.198]; SRMR = 0.147).
5) Adding the two residual autocorrelations between T2 and T3, and T3 and T4, constrained to be equal, to Model 4. This resulted in poor fit (χ²(8) = 63.99, p < .001; CFI = 0.942; TLI = 0.927; RMSEA = 0.138, 90% CI = [0.108, 0.170]; SRMR = 0.105).
6) **Loglinear model:** a model with two covarying growth factors, defining the slope factor loadings as the log of time in months. This resulted in poor fit (χ²(10) = 57.32, p < .001; CFI = 0.951; TLI = 0.951; RMSEA = 0.113, 90% CI = [0.086, 0.143]; SRMR = 0.071).
7) Adding the residual autocorrelation to model 6. This resulted in borderline acceptable fit (χ²(9) = 34.92, p < .001; CFI = 0.973; TLI = 0.970; RMSEA = 0.088, 90% CI = [0.059, 0.120]; SRMR = 0.046).
8) **Piecewise model:** a piecewise model with a random intercept, random slope from pretreatment to first follow-up (T1 to T2), and a fixed slope for the follow-up phase (T3 to T5). This model could not compute standard errors. Adding the residual autocorrelation resulted in warnings regarding non-positive definite covariance matrices for parameters and latent variables.
9) **Freed slope**: a model with two covarying growth factors, defining the first two slope factor loadings as 0 and 1, and allowing the rest to be estimated freely, so that the factor loadings following T2 are scaled to the amount change that happened between pretreatment and first follow-up. This model showed borderline acceptable fit (χ²(7) = 27.54, p < .001; CFI = 0.979; TLI = 0.970; RMSEA = 0.089, 90% CI = [0.056, 0.125]; SRMR = 0.034).
10) Adding the two residual autocorrelations between T2 and T3, and T3 and T4, constrained to be equal, to Model 9. This resulted in excellent fit (χ²(6) = 7.50, p = 0.277; CFI = 0.998; TLI = 0.997; RMSEA = 0.026, 90% CI = [0.000, 0.076]; SRMR = 0.020).

We considered two of these models. Taken together, we concluded that the loglinear model 7 had borderline acceptable fit, and an interpretable slope that matched the observed pattern of change when plotting the data with loess curves in the R package *ggplot*, suggesting a faster rate of initial change which then flattens out over time. The freed slope model 10 had excellent fit. Though harder to interpret, this slope might serve the purpose of detrending the data. The factor loadings were as follows: 0, 1, 0.953, 1.028, 1.059. Similar to the loglinear model, this slope suggests that the largest proportion of change happens from pretreatment to first follow-up (T2). There is then a slight dip at the next follow-up (T3), followed by continued gradual improvement (T4 and T5). In preparation for the multivariate LCM-SR, we added all autoregressive paths between the latent residuals in these two competing models, constraining the regression of T4 on T3 and T3 on T2 to be equal (in place of the previous autocorrelation in the models). In both cases, the additional parameters led to warnings of negative variance on the T5 latent residual. We then constrained the model further, constraining the within-person latent residual for each outcome to be equal at each timepoint after T1. For the freed slope, this still resulted in a negative latent variable variance. For the log slope, this model converged without warnings, and showed acceptable fit (χ²(10) = 30.76, p < .001; CFI = 0.978; TLI = 0.978; RMSEA = 0.075, 90% CI = [0.046, 0.106]; SRMR = 0.051). Consequently, we retained this loglinear LCM with the two equality-constrained autoregressions and four equality-constrained variances. The fixed factor loadings were the log of time in months as follows: 0, 2.79, 3.1, 3.34, 3.83.

### Quantity and Quality of Social Relationships

A linear growth model was retained for the Quantity and Quality of Social Relationships (QQSR), since this model showed excellent fit (χ²(10) = 12.95, p = 0.226; CFI = 0.997; TLI = 0.997; RMSEA = 0.028, 90% CI = [0.000, 0.067]; SRMR = 0.033) and has a readily interpretable slope. This was a significant improvement over an intercept-only model (Δχ²(3) = 40.24, p < .001). We added full autoregressive paths to this model. These additional parameters led to warnings of negative variance on the T5 latent residual. We further constrained the within-person latent residual for each outcome to be equal at each timepoint after T1. This model converged without warnings, and showed excellent fit (χ²(10) = 16.70, p = 0.081; CFI = 0.993; TLI = 0.993; RMSEA = 0.043, 90% CI = [0.000, 0.077]; SRMR = 0.051). Consequently, we retained this linear LCM with the two constrained autoregressions and four constrained variances. The fixed factor loadings were time in years as follows: 0, 1.36, 1.86, 2.36, 3.86.

## Multivariate Latent Curve Model with Structured Residuals (mLCM-SR)

We then combined the univariate models for Saff and QQSR, adding cross-lagged regression paths, and allowing all growth factors to covary. We constrained time-specific residual covariance to be equal after T1. We relaxed the constraints on the auto-regressive paths, so that the constraints were similar to the final RI-CLPM, making it a detrended version of the same model. This model showed excellent fit (χ²(29) = 52.40, p = 0.005; CFI = 0.988; TLI = 0.982; RMSEA = 0.047, 90% CI = [0.025, 0.067]; SRMR = 0.042).

As a sensitivity analysis, we added t1 personality disorder (PD) as a predictor of the intercepts and slopes. The model showed good fit (χ²(35) = 71.59, p < .001; CFI = 0.982; TLI = 0.972; RMSEA = 0.053, 90% CI = [0.036, 0.071]; SRMR = 0.038).

Model results are reported in the next section for the final RI-CLPM, the final LCM-SR, and the LCM-SR controlling for t1 PD. In models with Saff, one case was deleted due to missing data. In models with t1 PD, four cases were deleted due to missing diagnostic information.

# Model Results: RI-CLPM

| *Regressions: Random Intercept Cross-Lagged Panel Model (RI-CLPM)* | | | | | |
| --- | --- | --- | --- | --- | --- |
| Outcome | Predictor | b [95% CI] | *SE* | *Z* | *p* |
| wSaff_t2 | wSaff_t1 | -0.49 [-0.75, -0.23] | 0.13 | -3.73 | < .001*** |
| wSaff_t3 | wSaff_t2 | 0.56 [0.39, 0.72] | 0.08 | 6.56 | < .001*** |
| wSaff_t4 | wSaff_t3 | 0.63 [0.47, 0.78] | 0.08 | 8.07 | < .001*** |
| wSaff_t5 | wSaff_t4 | 0.63 [0.48, 0.78] | 0.08 | 8.14 | < .001*** |
| wTotalQQSR_t2 | wTotalQQSR_t1 | 0.16 [-0.03, 0.35] | 0.10 | 1.61 | .107 |
| wTotalQQSR_t3 | wTotalQQSR_t2 | 0.06 [-0.21, 0.33] | 0.14 | 0.46 | .643 |
| wTotalQQSR_t4 | wTotalQQSR_t3 | 0.22 [-0.08, 0.52] | 0.15 | 1.45 | .148 |
| wTotalQQSR_t5 | wTotalQQSR_t4 | 0.42 [0.22, 0.62] | 0.10 | 4.15 | < .001*** |
| wSaff_t2 | wTotalQQSR_t1 | 0.01 [-0.19, 0.21] | 0.10 | 0.07 | .942 |
| wSaff_t3 | wTotalQQSR_t2 | 0.34 [0.16, 0.52] | 0.09 | 3.78 | < .001*** |
| wSaff_t4 | wTotalQQSR_t3 | -0.02 [-0.21, 0.17] | 0.10 | -0.21 | .832 |
| wSaff_t5 | wTotalQQSR_t4 | 0.14 [-0.04, 0.32] | 0.09 | 1.54 | .124 |
| wTotalQQSR_t2 | wSaff_t1 | -0.26 [-0.48, -0.04] | 0.11 | -2.29 | .022* |
| wTotalQQSR_t3 | wSaff_t2 | 0.20 [-0.02, 0.42] | 0.11 | 1.81 | .070 |
| wTotalQQSR_t4 | wSaff_t3 | 0.31 [0.15, 0.47] | 0.08 | 3.84 | < .001*** |
| wTotalQQSR_t5 | wSaff_t4 | 0.08 [-0.08, 0.24] | 0.08 | 0.97 | .333 |
| *Note.* wTotalQQSR and wSaff = within-person latent residuals of the Quantity and Quality of Social Relationships and the Self-Affiliation dimension of the Structural Assessment of Social Behaviour (SASB), t1 = pretreatment, t2 = post treatment, t3 = six months post t2, t4 = one year post t2, t5 = two and a half years post t2. Autoregressive and cross-lagged regression coefficients with 95% Confidence Interval. Variances of each within-person latent variable, and their covariance at each wave, were constrained to be equal across waves T2 to T5. Therapist ID was entered to the cluster argument for cluster-robust standard errors. * = p < .05, ** = p < .01, *** = p < .001. | | | | | |

| *Standardised Regressions: Random Intercept Cross-Lagged Panel Model (RI-CLPM)* | | | | | | |
| --- | --- | --- | --- | --- | --- | --- |
| Outcome | op | Predictor | se | *z* | *p* | b* [95% CI] |
| wSaff_t2 | ~ | wSaff_t1 | 0.09 | -5.23 | < .001*** | -0.49 [-0.68, -0.31] |
| wSaff_t3 | ~ | wSaff_t2 | 0.07 | 7.57 | < .001*** | 0.49 [0.37, 0.62] |
| wSaff_t4 | ~ | wSaff_t3 | 0.06 | 11.24 | < .001*** | 0.63 [0.52, 0.74] |
| wSaff_t5 | ~ | wSaff_t4 | 0.06 | 10.11 | < .001*** | 0.61 [0.49, 0.72] |
| wTotalQQSR_t2 | ~ | wTotalQQSR_t1 | 0.11 | 1.59 | .111 | 0.18 [-0.04, 0.40] |
| wTotalQQSR_t3 | ~ | wTotalQQSR_t2 | 0.14 | 0.46 | .643 | 0.06 [-0.21, 0.34] |
| wTotalQQSR_t4 | ~ | wTotalQQSR_t3 | 0.14 | 1.47 | .140 | 0.20 [-0.07, 0.47] |
| wTotalQQSR_t5 | ~ | wTotalQQSR_t4 | 0.09 | 4.64 | < .001*** | 0.42 [0.24, 0.60] |
| wSaff_t2 | ~ | wTotalQQSR_t1 | 0.10 | 0.07 | .942 | 0.01 [-0.20, 0.21] |
| wSaff_t3 | ~ | wTotalQQSR_t2 | 0.07 | 3.89 | < .001*** | 0.27 [0.13, 0.40] |
| wSaff_t4 | ~ | wTotalQQSR_t3 | 0.08 | -0.21 | .831 | -0.02 [-0.17, 0.13] |
| wSaff_t5 | ~ | wTotalQQSR_t4 | 0.07 | 1.56 | .118 | 0.12 [-0.03, 0.26] |
| wTotalQQSR_t2 | ~ | wSaff_t1 | 0.11 | -2.75 | .006** | -0.29 [-0.50, -0.08] |
| wTotalQQSR_t3 | ~ | wSaff_t2 | 0.12 | 1.87 | .061 | 0.23 [-0.01, 0.47] |
| wTotalQQSR_t4 | ~ | wSaff_t3 | 0.10 | 3.75 | < .001*** | 0.36 [0.17, 0.55] |
| wTotalQQSR_t5 | ~ | wSaff_t4 | 0.10 | 0.97 | .333 | 0.09 [-0.09, 0.28] |

| *Covariances: Random Intercept Cross-Lagged Panel Model (RI-CLPM)* | | | | | | | | |
| --- | --- | --- | --- | --- | --- | --- | --- | --- |
| Variable 1 | Variable 2 | *SE* | *Z* | *p* | σ | 95% CI (σ) | *r* | 95% CI (*r*) |
| Int_Saff | Int_QQSR | 0.01 | 6.88 | < .001*** | 0.06 | [0.04, 0.08] | .44 | [0.34, 0.53] |
| wSaff_t1 | wTotalQQSR_t1 | 0.01 | 1.40 | .160 | 0.01 | [-0.01, 0.03] | .17 | [-0.05, 0.39] |
| wSaff_t2 | wTotalQQSR_t2 | 0.00 | 4.27 | < .001*** | 0.01 | [0.01, 0.02] | .23 | [0.13, 0.33] |
| wSaff_t3 | wTotalQQSR_t3 | 0.00 | 4.27 | < .001*** | 0.01 | [0.01, 0.02] | .23 | [0.13, 0.33] |
| wSaff_t4 | wTotalQQSR_t4 | 0.00 | 4.27 | < .001*** | 0.01 | [0.01, 0.02] | .23 | [0.13, 0.33] |
| wSaff_t5 | wTotalQQSR_t5 | 0.00 | 4.27 | < .001*** | 0.01 | [0.01, 0.02] | .23 | [0.13, 0.33] |
| *Note.* wTotalQQSR and wSaff = within-person latent residuals of the Quantity and Quality of Social Relationships and the Self-Affiliation dimension of the Structural Assessment of Social Behaviour (SASB), t1 = pretreatment, t2 = post treatment, t3 = six months post t2, t4 = one year post t2, t5 = two and a half years post t2. Variances of each within-person latent variable, and their covariance at each wave, were constrained to be equal across waves T2 to T5. Therapist ID was entered to the cluster argument for cluster-robust standard errors. * = p < .05, ** = p < .01, *** = p < .001. | | | | | | | | |

| *Variances: Random Intercept Cross-Lagged Panel Model (RI-CLPM)* | | | |
| --- | --- | --- | --- |
| Variable | *p* | σ^2^ | 95% CI |
| Int_Saff | < .001*** | 0.15 | [0.13, 0.17] |
| Int_QQSR | < .001*** | 0.13 | [0.11, 0.16] |
| wSaff_t1 | < .001*** | 0.08 | [0.06, 0.11] |
| wSaff_t2 | < .001*** | 0.06 | [0.05, 0.07] |
| wSaff_t3 | < .001*** | 0.06 | [0.05, 0.07] |
| wSaff_t4 | < .001*** | 0.06 | [0.05, 0.07] |
| wSaff_t5 | < .001*** | 0.06 | [0.05, 0.07] |
| wTotalQQSR_t1 | < .001*** | 0.09 | [0.07, 0.11] |
| wTotalQQSR_t2 | < .001*** | 0.06 | [0.05, 0.07] |
| wTotalQQSR_t3 | < .001*** | 0.06 | [0.05, 0.07] |
| wTotalQQSR_t4 | < .001*** | 0.06 | [0.05, 0.07] |
| wTotalQQSR_t5 | < .001*** | 0.06 | [0.05, 0.07] |
| *Note.* wTotalQQSR and wSaff = within-person latent residuals of the Quantity and Quality of Social Relationships and the Self-Affiliation dimension of the Structural Assessment of Social Behaviour (SASB), t1 = pretreatment, t2 = post treatment, t3 = six months post t2, t4 = one year post t2, t5 = two and a half years post t2. Variances of each within-person latent variable, and their covariance at each wave, were constrained to be equal across waves T2 to T5. Therapist ID was entered to the cluster argument for cluster-robust standard errors. * = p < .05, ** = p < .01, *** = p < .001. | | | |

| *Intercepts: Random Intercept Cross-Lagged Panel Model (RI-CLPM)* | | | | |
| --- | --- | --- | --- | --- |
| Variable | *SE* | *Z* | *p* | b [95% CI] |
| Saff_t1 | 0.02 | 102.87 | < .001*** | 2.54 [2.49, 2.59] |
| Saff_t2 | 0.03 | 95.96 | < .001*** | 2.90 [2.84, 2.96] |
| Saff_t3 | 0.03 | 99.14 | < .001*** | 2.89 [2.83, 2.94] |
| Saff_t4 | 0.03 | 89.92 | < .001*** | 2.90 [2.84, 2.97] |
| Saff_t5 | 0.04 | 82.53 | < .001*** | 2.92 [2.85, 2.99] |
| TotalQQSR_t1 | 0.03 | 104.74 | < .001*** | 2.68 [2.63, 2.73] |
| TotalQQSR_t2 | 0.03 | 107.34 | < .001*** | 2.71 [2.66, 2.76] |
| TotalQQSR_t3 | 0.03 | 96.14 | < .001*** | 2.72 [2.67, 2.78] |
| TotalQQSR_t4 | 0.03 | 96.73 | < .001*** | 2.71 [2.66, 2.77] |
| TotalQQSR_t5 | 0.03 | 97.06 | < .001*** | 2.75 [2.70, 2.81] |
| *Note.* wTotalQQSR and wSaff = within-person latent residuals of the Quantity and Quality of Social Relationships and the Self-Affiliation dimension of the Structural Assessment of Social Behaviour (SASB), t1 = pretreatment, t2 = post treatment, t3 = six months post t2, t4 = one year post t2, t5 = two and a half years post t2. Int = random intercept. Variances of each within-person latent variable, and their covariance at each wave, were constrained to be equal across waves T2 to T5. Therapist ID was entered to the cluster argument for cluster-robust standard errors. * = p < .05, ** = p < .01, *** = p < .001. | | | | |

# Model Results: LCM-SR

| *Regressions: Latent Curve Model with Structured Residuals (LCM-SR)* | | | | | |
| --- | --- | --- | --- | --- | --- |
| Outcome | Predictor | b [95% CI] | *SE* | *Z* | *p* |
| wSaff_t2 | wSaff_t1 | -0.72 [-1.43, -0.00] | 0.36 | -1.97 | .049* |
| wSaff_t3 | wSaff_t2 | 0.40 [0.18, 0.63] | 0.11 | 3.51 | < .001*** |
| wSaff_t4 | wSaff_t3 | 0.46 [0.26, 0.65] | 0.10 | 4.60 | < .001*** |
| wSaff_t5 | wSaff_t4 | 0.37 [0.11, 0.63] | 0.13 | 2.79 | .005** |
| wTotalQQSR_t2 | wTotalQQSR_t1 | 0.25 [-0.06, 0.56] | 0.16 | 1.59 | .111 |
| wTotalQQSR_t3 | wTotalQQSR_t2 | 0.05 [-0.21, 0.30] | 0.13 | 0.36 | .716 |
| wTotalQQSR_t4 | wTotalQQSR_t3 | 0.19 [-0.11, 0.48] | 0.15 | 1.24 | .216 |
| wTotalQQSR_t5 | wTotalQQSR_t4 | 0.38 [0.20, 0.56] | 0.09 | 4.09 | < .001*** |
| wSaff_t2 | wTotalQQSR_t1 | 0.21 [-0.20, 0.61] | 0.21 | 0.98 | .325 |
| wSaff_t3 | wTotalQQSR_t2 | 0.36 [0.15, 0.57] | 0.11 | 3.33 | .001*** |
| wSaff_t4 | wTotalQQSR_t3 | -0.10 [-0.28, 0.08] | 0.09 | -1.09 | .275 |
| wSaff_t5 | wTotalQQSR_t4 | 0.14 [-0.01, 0.30] | 0.08 | 1.80 | .072 |
| wTotalQQSR_t2 | wSaff_t1 | -0.31 [-0.87, 0.25] | 0.28 | -1.09 | .275 |
| wTotalQQSR_t3 | wSaff_t2 | 0.10 [-0.09, 0.28] | 0.09 | 1.01 | .311 |
| wTotalQQSR_t4 | wSaff_t3 | 0.30 [0.11, 0.49] | 0.10 | 3.13 | .002** |
| wTotalQQSR_t5 | wSaff_t4 | 0.03 [-0.18, 0.24] | 0.11 | 0.30 | .766 |
| *Note.* wTotalQQSR and wSaff = within-person latent residuals of the Quantity and Quality of Social Relationships and the Self-Affiliation dimension of the Structural Assessment of Social Behaviour (SASB), t1 = pretreatment, t2 = post treatment, t3 = six months post t2, t4 = one year post t2, t5 = two and a half years post t2. Autoregressive and cross-lagged regression coefficients with 95% Confidence Interval. Variances of each within-person latent variable, and their covariance at each wave, were constrained to be equal across waves T2 to T5. Therapist ID was entered to the cluster argument for cluster-robust standard errors. * = p < .05, ** = p < .01, *** = p < .001. | | | | | |

| *Standardised Regressions: Latent Curve Model with Structured Residuals (LCM-SR)* | | | | | | |
| --- | --- | --- | --- | --- | --- | --- |
| Outcome | op | Predictor | se | *z* | *p* | b* [95% CI] |
| wSaff_t2 | ~ | wSaff_t1 | 0.16 | -3.66 | < .001*** | -0.58 [-0.89, -0.27] |
| wSaff_t3 | ~ | wSaff_t2 | 0.10 | 3.92 | < .001*** | 0.38 [0.19, 0.58] |
| wSaff_t4 | ~ | wSaff_t3 | 0.10 | 4.98 | < .001*** | 0.49 [0.30, 0.68] |
| wSaff_t5 | ~ | wSaff_t4 | 0.12 | 3.04 | .002** | 0.38 [0.13, 0.62] |
| wTotalQQSR_t2 | ~ | wTotalQQSR_t1 | 0.19 | 1.62 | .105 | 0.30 [-0.06, 0.67] |
| wTotalQQSR_t3 | ~ | wTotalQQSR_t2 | 0.14 | 0.36 | .717 | 0.05 [-0.22, 0.31] |
| wTotalQQSR_t4 | ~ | wTotalQQSR_t3 | 0.14 | 1.26 | .207 | 0.17 [-0.10, 0.44] |
| wTotalQQSR_t5 | ~ | wTotalQQSR_t4 | 0.08 | 4.57 | < .001*** | 0.38 [0.22, 0.55] |
| wSaff_t2 | ~ | wTotalQQSR_t1 | 0.22 | 1.03 | .302 | 0.23 [-0.21, 0.67] |
| wSaff_t3 | ~ | wTotalQQSR_t2 | 0.08 | 4.03 | < .001*** | 0.32 [0.17, 0.48] |
| wSaff_t4 | ~ | wTotalQQSR_t3 | 0.08 | -1.11 | .268 | -0.09 [-0.25, 0.07] |
| wSaff_t5 | ~ | wTotalQQSR_t4 | 0.08 | 1.76 | .079 | 0.15 [-0.02, 0.31] |
| wTotalQQSR_t2 | ~ | wSaff_t1 | 0.21 | -1.31 | .189 | -0.27 [-0.67, 0.13] |
| wTotalQQSR_t3 | ~ | wSaff_t2 | 0.10 | 1.01 | .311 | 0.11 [-0.10, 0.31] |
| wTotalQQSR_t4 | ~ | wSaff_t3 | 0.11 | 3.00 | .003** | 0.32 [0.11, 0.53] |
| wTotalQQSR_t5 | ~ | wSaff_t4 | 0.11 | 0.30 | .765 | 0.03 [-0.18, 0.24] |

| *Covariances: Latent Curve Model with Structured Residuals (LCM-SR)* | | | | | | | | |
| --- | --- | --- | --- | --- | --- | --- | --- | --- |
| Variable 1 | Variable 2 | *SE* | *Z* | *p* | σ | 95% CI (σ) | *r* | 95% CI (*r*) |
| Saff_int | Saff_slp | 0.01 | -1.59 | .111 | -0.01 | [-0.03, 0.00] | -.31 | [-0.57, -0.04] |
| QQSR_int | QQSR_slp | 0.00 | 0.94 | .348 | 0.00 | [-0.00, 0.01] | .43 | [-1.26, 2.12] |
| Saff_int | QQSR_int | 0.02 | 2.91 | .004** | 0.05 | [0.01, 0.08] | .31 | [0.12, 0.50] |
| Saff_slp | QQSR_int | 0.00 | 2.03 | .042* | 0.01 | [0.00, 0.02] | .25 | [0.00, 0.49] |
| Saff_int | QQSR_slp | 0.00 | 0.51 | .610 | 0.00 | [-0.01, 0.01] | .22 | [-0.73, 1.17] |
| Saff_slp | QQSR_slp | 0.00 | -0.10 | .919 | -0.00 | [-0.00, 0.00] | -.06 | [-1.31, 1.19] |
| wSaff_t1 | wTotalQQSR_t1 | 0.02 | 1.98 | .048* | 0.03 | [0.00, 0.06] | .49 | [0.03, 0.95] |
| wSaff_t2 | wTotalQQSR_t2 | 0.00 | 2.71 | .007** | 0.01 | [0.00, 0.02] | .22 | [0.07, 0.36] |
| wSaff_t3 | wTotalQQSR_t3 | 0.00 | 2.71 | .007** | 0.01 | [0.00, 0.02] | .22 | [0.07, 0.36] |
| wSaff_t4 | wTotalQQSR_t4 | 0.00 | 2.71 | .007** | 0.01 | [0.00, 0.02] | .22 | [0.07, 0.36] |
| wSaff_t5 | wTotalQQSR_t5 | 0.00 | 2.71 | .007** | 0.01 | [0.00, 0.02] | .22 | [0.07, 0.36] |
| *Note.* wTotalQQSR and wSaff = within-person latent residuals of the Quantity and Quality of Social Relationships and the Self-Affiliation dimension of the Structural Assessment of Social Behaviour (SASB), t1 = pretreatment, t2 = post treatment, t3 = six months post t2, t4 = one year post t2, t5 = two and a half years post t2. Variances of each within-person latent variable, and their covariance at each wave, were constrained to be equal across waves T2 to T5. Therapist ID was entered to the cluster argument for cluster-robust standard errors. * = p < .05, ** = p < .01, *** = p < .001. | | | | | | | | |

| *Variances: Latent Curve Model with Structured Residuals (LCM-SR)* | | | |
| --- | --- | --- | --- |
| Variable | *p* | σ^2^ | 95% CI |
| wSaff_t1 | .042* | 0.05 | [0.00, 0.09] |
| wSaff_t2 | < .001*** | 0.05 | [0.04, 0.06] |
| wSaff_t3 | < .001*** | 0.05 | [0.04, 0.06] |
| wSaff_t4 | < .001*** | 0.05 | [0.04, 0.06] |
| wSaff_t5 | < .001*** | 0.05 | [0.04, 0.06] |
| wTotalQQSR_t1 | < .001*** | 0.09 | [0.06, 0.12] |
| wTotalQQSR_t2 | < .001*** | 0.06 | [0.05, 0.07] |
| wTotalQQSR_t3 | < .001*** | 0.06 | [0.05, 0.07] |
| wTotalQQSR_t4 | < .001*** | 0.06 | [0.05, 0.07] |
| wTotalQQSR_t5 | < .001*** | 0.06 | [0.05, 0.07] |
| Saff_int | < .001*** | 0.18 | [0.13, 0.22] |
| Saff_slp | .001** | 0.01 | [0.00, 0.01] |
| QQSR_int | < .001*** | 0.12 | [0.09, 0.15] |
| QQSR_slp | .645 | 0.00 | [-0.00, 0.00] |
| *Note.* wTotalQQSR and wSaff = within-person latent residuals of the Quantity and Quality of Social Relationships and the Self-Affiliation dimension of the Structural Assessment of Social Behaviour (SASB), t1 = pretreatment, t2 = post treatment, t3 = six months post t2, t4 = one year post t2, t5 = two and a half years post t2. Variances of each within-person latent variable, and their covariance at each wave, were constrained to be equal across waves T2 to T5. Therapist ID was entered to the cluster argument for cluster-robust standard errors. * = p < .05, ** = p < .01, *** = p < .001. | | | |

| *Intercepts: Latent Curve Model with Structured Residuals (LCM-SR)* | | | | |
| --- | --- | --- | --- | --- |
| Variable | *SE* | *Z* | *p* | b [95% CI] |
| Saff_int | 0.02 | 111.51 | < .001*** | 2.56 [2.51, 2.60] |
| Saff_slp | 0.01 | 12.52 | < .001*** | 0.10 [0.09, 0.12] |
| QQSR_int | 0.02 | 114.05 | < .001*** | 2.68 [2.64, 2.73] |
| QQSR_slp | 0.01 | 3.07 | .002** | 0.02 [0.01, 0.03] |
| *Note.* wTotalQQSR and wSaff = within-person latent residuals of the Quantity and Quality of Social Relationships and the Self-Affiliation dimension of the Structural Assessment of Social Behaviour (SASB), t1 = pretreatment, t2 = post treatment, t3 = six months post t2, t4 = one year post t2, t5 = two and a half years post t2. Int = random intercept. Variances of each within-person latent variable, and their covariance at each wave, were constrained to be equal across waves T2 to T5. Therapist ID was entered to the cluster argument for cluster-robust standard errors.In LCM-SR, time was coded in months for QQSR and in the log of months for Saff. * = p < .05, ** = p < .01, *** = p < .001. | | | | |

# Model Results: LCM-SR Controlling for Personality Disorder

| *Regressions: LCM-SR Controlling for Personality Disorder* | | | | | |
| --- | --- | --- | --- | --- | --- |
| Outcome | Predictor | b [95% CI] | *SE* | *Z* | *p* |
| wSaff_t2 | wSaff_t1 | -1.05 [-2.06, -0.05] | 0.51 | -2.06 | .039* |
| wSaff_t3 | wSaff_t2 | 0.41 [0.19, 0.63] | 0.11 | 3.62 | < .001*** |
| wSaff_t4 | wSaff_t3 | 0.46 [0.26, 0.66] | 0.10 | 4.48 | < .001*** |
| wSaff_t5 | wSaff_t4 | 0.36 [0.11, 0.61] | 0.13 | 2.84 | .005** |
| wTotalQQSR_t2 | wTotalQQSR_t1 | 0.33 [-0.04, 0.69] | 0.19 | 1.77 | .077 |
| wTotalQQSR_t3 | wTotalQQSR_t2 | 0.05 [-0.20, 0.31] | 0.13 | 0.42 | .677 |
| wTotalQQSR_t4 | wTotalQQSR_t3 | 0.18 [-0.11, 0.47] | 0.15 | 1.18 | .236 |
| wTotalQQSR_t5 | wTotalQQSR_t4 | 0.39 [0.20, 0.57] | 0.09 | 4.08 | < .001*** |
| wSaff_t2 | wTotalQQSR_t1 | 0.33 [-0.18, 0.84] | 0.26 | 1.26 | .207 |
| wSaff_t3 | wTotalQQSR_t2 | 0.34 [0.13, 0.54] | 0.10 | 3.23 | .001** |
| wSaff_t4 | wTotalQQSR_t3 | -0.09 [-0.26, 0.09] | 0.09 | -0.97 | .330 |
| wSaff_t5 | wTotalQQSR_t4 | 0.14 [-0.01, 0.30] | 0.08 | 1.78 | .075 |
| wTotalQQSR_t2 | wSaff_t1 | -0.53 [-1.30, 0.24] | 0.39 | -1.35 | .176 |
| wTotalQQSR_t3 | wSaff_t2 | 0.14 [-0.05, 0.32] | 0.09 | 1.45 | .146 |
| wTotalQQSR_t4 | wSaff_t3 | 0.30 [0.11, 0.49] | 0.10 | 3.07 | .002** |
| wTotalQQSR_t5 | wSaff_t4 | 0.02 [-0.18, 0.23] | 0.10 | 0.20 | .840 |
| Saff_int | t1PD | -0.31 [-0.41, -0.22] | 0.05 | -6.75 | < .001*** |
| Saff_slp | t1PD | 0.02 [-0.01, 0.05] | 0.02 | 1.37 | .171 |
| QQSR_int | t1PD | -0.23 [-0.31, -0.14] | 0.04 | -5.39 | < .001*** |
| QQSR_slp | t1PD | 0.02 [-0.01, 0.04] | 0.01 | 1.24 | .214 |
| *Note.* wTotalQQSR and wSaff = within-person latent residuals of the Quantity and Quality of Social Relationships and the Self-Affiliation dimension of the Structural Assessment of Social Behaviour (SASB), t1 = pretreatment, t2 = post treatment, t3 = six months post t2, t4 = one year post t2, t5 = two and a half years post t2. Autoregressive and cross-lagged regression coefficients with 95% Confidence Interval. Variances of each within-person latent variable, and their covariance at each wave, were constrained to be equal across waves T2 to T5. Therapist ID was entered to the cluster argument for cluster-robust standard errors. * = p < .05, ** = p < .01, *** = p < .001. t1PD = pretreatment personality disorder (coded as 0 = none, 1 = present) | | | | | |

| *Covariances: LCM-SR Controlling for Personality Disorder* | | | | | | | | |
| --- | --- | --- | --- | --- | --- | --- | --- | --- |
| Variable 1 | Variable 2 | *SE* | *Z* | *p* | σ | 95% CI (σ) | *r* | 95% CI (*r*) |
| Saff_int | Saff_slp | 0.01 | -2.55 | .011* | -0.01 | [-0.03, -0.00] | -.36 | [-0.55, -0.17] |
| QQSR_int | QQSR_slp | 0.00 | 1.15 | .248 | 0.01 | [-0.00, 0.01] | .59 | [-1.47, 2.64] |
| Saff_int | QQSR_int | 0.01 | 1.93 | .054 | 0.03 | [-0.00, 0.06] | .21 | [0.01, 0.42] |
| Saff_slp | QQSR_int | 0.00 | 2.12 | .034* | 0.01 | [0.00, 0.02] | .26 | [0.02, 0.49] |
| Saff_int | QQSR_slp | 0.00 | 0.62 | .533 | 0.00 | [-0.01, 0.01] | .27 | [-0.70, 1.23] |
| Saff_slp | QQSR_slp | 0.00 | 0.15 | .878 | 0.00 | [-0.00, 0.00] | .08 | [-0.97, 1.13] |
| wSaff_t1 | wTotalQQSR_t1 | 0.01 | 1.91 | .056 | 0.03 | [-0.00, 0.06] | .53 | [0.03, 1.02] |
| wSaff_t2 | wTotalQQSR_t2 | 0.00 | 2.55 | .011* | 0.01 | [0.00, 0.02] | .21 | [0.06, 0.35] |
| wSaff_t3 | wTotalQQSR_t3 | 0.00 | 2.55 | .011* | 0.01 | [0.00, 0.02] | .21 | [0.06, 0.35] |
| wSaff_t4 | wTotalQQSR_t4 | 0.00 | 2.55 | .011* | 0.01 | [0.00, 0.02] | .21 | [0.06, 0.35] |
| wSaff_t5 | wTotalQQSR_t5 | 0.00 | 2.55 | .011* | 0.01 | [0.00, 0.02] | .21 | [0.06, 0.35] |
| *Note.* wTotalQQSR and wSaff = within-person latent residuals of the Quantity and Quality of Social Relationships and the Self-Affiliation dimension of the Structural Assessment of Social Behaviour (SASB), t1 = pretreatment, t2 = post treatment, t3 = six months post t2, t4 = one year post t2, t5 = two and a half years post t2. Variances of each within-person latent variable, and their covariance at each wave, were constrained to be equal across waves T2 to T5. Therapist ID was entered to the cluster argument for cluster-robust standard errors. * = p < .05, ** = p < .01, *** = p < .001. | | | | | | | | |

| *Variances: LCM-SR Controlling for Personality Disorder* | | | |
| --- | --- | --- | --- |
| Variable | *p* | σ^2^ | 95% CI |
| wSaff_t1 | .067 | 0.03 | [-0.00, 0.07] |
| wSaff_t2 | < .001*** | 0.05 | [0.04, 0.06] |
| wSaff_t3 | < .001*** | 0.05 | [0.04, 0.06] |
| wSaff_t4 | < .001*** | 0.05 | [0.04, 0.06] |
| wSaff_t5 | < .001*** | 0.05 | [0.04, 0.06] |
| wTotalQQSR_t1 | < .001*** | 0.09 | [0.06, 0.12] |
| wTotalQQSR_t2 | < .001*** | 0.06 | [0.05, 0.07] |
| wTotalQQSR_t3 | < .001*** | 0.06 | [0.05, 0.07] |
| wTotalQQSR_t4 | < .001*** | 0.06 | [0.05, 0.07] |
| wTotalQQSR_t5 | < .001*** | 0.06 | [0.05, 0.07] |
| Saff_int | < .001*** | 0.16 | [0.13, 0.20] |
| Saff_slp | < .001*** | 0.01 | [0.01, 0.02] |
| QQSR_int | < .001*** | 0.11 | [0.08, 0.14] |
| QQSR_slp | .630 | 0.00 | [-0.00, 0.00] |
| *Note.* wTotalQQSR and wSaff = within-person latent residuals of the Quantity and Quality of Social Relationships and the Self-Affiliation dimension of the Structural Assessment of Social Behaviour (SASB), t1 = pretreatment, t2 = post treatment, t3 = six months post t2, t4 = one year post t2, t5 = two and a half years post t2. Variances of each within-person latent variable, and their covariance at each wave, were constrained to be equal across waves T2 to T5. Therapist ID was entered to the cluster argument for cluster-robust standard errors. * = p < .05, ** = p < .01, *** = p < .001. | | | |

| *Intercepts: LCM-SR Controlling for Personality Disorder* | | | | |
| --- | --- | --- | --- | --- |
| Variable | *SE* | *Z* | *p* | b [95% CI] |
| Saff_int | 0.07 | 42.66 | < .001*** | 3.04 [2.90, 3.18] |
| Saff_slp | 0.03 | 2.78 | .005** | 0.07 [0.02, 0.12] |
| QQSR_int | 0.06 | 47.07 | < .001*** | 3.03 [2.90, 3.15] |
| QQSR_slp | 0.02 | -0.25 | .803 | -0.00 [-0.04, 0.03] |
| *Note.* wTotalQQSR and wSaff = within-person latent residuals of the Quantity and Quality of Social Relationships and the Self-Affiliation dimension of the Structural Assessment of Social Behaviour (SASB), t1 = pretreatment, t2 = post treatment, t3 = six months post t2, t4 = one year post t2, t5 = two and a half years post t2. Int = random intercept. Variances of each within-person latent variable, and their covariance at each wave, were constrained to be equal across waves T2 to T5. Therapist ID was entered to the cluster argument for cluster-robust standard errors.In LCM-SR, time was coded in months for QQSR and in the log of months for Saff. * = p < .05, ** = p < .01, *** = p < .001. | | | | |
