# Supplementary material for: Connected to Others and Kind to Self: Social Connection and Self‐Affiliation Change in Open‐Ended Psychotherapy
Source: J Clin Psychol. 2026 Sep 4;82(12):2060–9. doi: 10.1002/jclp.70220 (PMC13733646; doi:10.1002/jclp.70220)
Supplement: Supplementary file 3 [file JCLP-82-2060-s003.docx]

### Supplement 01

### Descriptive Statistics

| **Table** | | | | | | |
| --- | --- | --- | --- | --- | --- | --- |
| *Quantity and Quality of Social Relationships Means and Standard Errors* | | | | | | |
| Index (range) | t1 | t2 | t3 | t4 | t5 | t6 |
| Total (1–4) | 2.68 (0.02) | 2.71 (0.03) | 2.71 (0.03) | 2.71 (0.03) | 2.76 (0.03) | 2.77 (0.10) |
| Contact (1–6) | 4.30 (0.04) | 4.32 (0.04) | 4.35 (0.05) | 4.29 (0.05) | 4.36 (0.04) | 4.47 (0.14) |
| Faffect (1–5) | 3.25 (0.04) | 3.31 (0.05) | 3.30 (0.05) | 3.29 (0.05) | 3.34 (0.05) | 3.43 (0.16) |
| Raffect (1–5) | 3.29 (0.04) | 3.29 (0.05) | 3.27 (0.05) | 3.32 (0.05) | 3.36 (0.05) | 3.43 (0.14) |
| Support (1–4) | 2.36 (0.03) | 2.43 (0.04) | 2.43 (0.04) | 2.45 (0.04) | 2.49 (0.04) | 2.37 (0.17) |
| FamOri (1–4) | 2.52 (0.03) | 2.52 (0.04) | 2.49 (0.04) | 2.52 (0.04) | 2.54 (0.04) | 2.53 (0.14) |
| CurFam (1–4) | 3.48 (0.03) | 3.48 (0.03) | 3.54 (0.03) | 3.53 (0.03) | 3.52 (0.03) | 3.53 (0.09) |
| OutFam (1–4) | 2.49 (0.03) | 2.53 (0.03) | 2.54 (0.04) | 2.54 (0.04) | 2.59 (0.03) | 2.63 (0.12) |
| NQQSR (0–9) | 8.32 (0.08) | 8.22 (0.10) | 8.19 (0.11) | 8.23 (0.10) | 8.27 (0.10) | 7.82 (0.40) |
| NFamOri (0–4) | 3.45 (0.04) | 3.34 (0.05) | 3.26 (0.07) | 3.22 (0.06) | 3.24 (0.06) | 2.90 (0.18) |
| NCurFam (0–2) | 1.43 (0.04) | 1.52 (0.04) | 1.50 (0.05) | 1.46 (0.05) | 1.55 (0.04) | 1.50 (0.14) |
| NOutFam (0–4) | 3.47 (0.04) | 3.44 (0.05) | 3.47 (0.06) | 3.52 (0.05) | 3.47 (0.05) | 3.63 (0.16) |
| *Note:* N patients responding to QQSR (Quantity and Quality of Social Relationships) per timepoint = t1: 367, t2: 293, t3: 224, t4: 231, t5: 256, t6: 23. QQSR abbreviations: Contact: frequency of contact, Faffect: freely expressed affection, Raffect: received affection, FamOri: family of origin (mother, father, sibling, other relation), CurFam: current family (partner and child), OutFam: outside family (friend, colleagues, neighbours). N: number of relationships present. t1: pretreatment, t2: post treatment, t3: six months post t2, t4: one year post t2, t5: two and a half years post t2, t6: six years post t2.10 | | | | | | |

| **Table** | | | | | | | | | | |
| --- | --- | --- | --- | --- | --- | --- | --- | --- | --- | --- |
| *Continuous Pretreatment Characteristics* | | | | | | | | | | |
| vars | *n* | mean | sd | median | min | max | range | skew | kurtosis | se |
| age | 370.00 | 34.89 | 9.43 | 34.00 | 18.00 | 65.00 | 47.00 | 0.54 | -0.21 | 0.49 |
| n_children | 356.00 | 1.48 | 1.19 | 2.00 | 0.00 | 5.00 | 5.00 | 0.23 | -0.78 | 0.06 |
| n_siblings | 364.00 | 3.09 | 1.52 | 3.00 | 0.00 | 10.00 | 10.00 | 1.23 | 2.71 | 0.08 |
| treatment_sessions | 363.00 | 51.74 | 59.39 | 35.00 | 0.00 | 364.00 | 364.00 | 2.70 | 8.50 | 3.12 |
| treatment_months | 366.00 | 20.62 | 19.65 | 13.82 | 0.00 | 97.70 | 97.70 | 1.75 | 2.79 | 1.03 |

| **Table** | | | |
| --- | --- | --- | --- |
| *Categorical Pretreatment Characteristics* | | | |
|  |  |  | Statistic (N=370) |
| gender | Man |  | 108 (29.2%) |
|  | Woman |  | 262 (70.8%) |
| marital_status | Cohabiting |  | 71 (19.2%) |
|  | Married |  | 127 (34.3%) |
|  | Separated/Divorced |  | 55 (14.9%) |
|  | Single |  | 108 (29.2%) |
|  | Widowed |  | 3 (0.8%) |
|  | Missing |  | 6 (1.6%) |
| education | Primary |  | 15 (4.1%) |
|  | Secondary |  | 49 (13.2%) |
|  | Vocational |  | 75 (20.3%) |
|  | High school |  | 83 (22.4%) |
|  | University/college |  | 139 (37.6%) |
|  | Missing |  | 9 (2.4%) |
| occupational_situation | Disability benefit |  | 9 (2.4%) |
|  | Sick leave |  | 101 (27.3%) |
|  | Stay at home parent |  | 12 (3.2%) |
|  | Student |  | 32 (8.6%) |
|  | Unemployed |  | 16 (4.3%) |
|  | Work rehabilitation |  | 32 (8.6%) |
|  | Working |  | 164 (44.3%) |
|  | Missing |  | 4 (1.1%) |
| money_difficulties | Enough |  | 216 (58.4%) |
|  | Usually enough |  | 67 (18.1%) |
|  | Half the time not enough |  | 32 (8.6%) |
|  | Usually not enough |  | 25 (6.8%) |
|  | Great difficulty |  | 20 (5.4%) |
|  | Missing |  | 10 (2.7%) |
| financial_childhood | Worse |  | 85 (23.0%) |
|  | Normal |  | 248 (67.0%) |
|  | Better |  | 30 (8.1%) |
|  | Missing |  | 7 (1.9%) |
| urban_origin | Countryside |  | 158 (42.7%) |
|  | Village |  | 75 (20.3%) |
|  | Small town |  | 58 (15.7%) |
|  | Larger city |  | 72 (19.5%) |
|  | Missing |  | 7 (1.9%) |
| minority_origin | Minority |  | 85 (23.0%) |
|  | Not minority |  | 276 (74.6%) |
|  | Missing |  | 9 (2.4%) |
| PD | NoPD |  | 171 (46.2%) |
|  | PD |  | 195 (52.7%) |
|  | Missing |  | 4 (1.1%) |
| *Note:* PD = Personality Disorder pretreatment. | | | |

| **Table** | | |
| --- | --- | --- |
| *Frequency of DSM-IV Axis I Disorders at T1* | | |
| Diagnosis | Frequency | % |
| MDD | 117 | 31.62 |
| Social | 97 | 26.22 |
| GAD | 82 | 22.16 |
| Dysthymia | 66 | 17.84 |
| Panic | 66 | 17.84 |
| Pain | 64 | 17.30 |
| Phobia | 53 | 14.32 |
| OCD | 46 | 12.43 |
| PTSD | 31 | 8.38 |
| Somatisation | 24 | 6.49 |
| Anx_IEC | 23 | 6.22 |
| Agora | 22 | 5.95 |
| Dep_IEC | 21 | 5.68 |
| BN | 17 | 4.59 |
| BodyDysmorphia | 15 | 4.05 |
| Adaptive | 14 | 3.78 |
| Overeating | 11 | 2.97 |
| Hypochondria | 8 | 2.16 |
| Somatoform_undiff | 7 | 1.89 |
| Alcohol | 5 | 1.35 |
| Other | 5 | 1.35 |
| BP1 | 4 | 1.08 |
| AN | 4 | 1.08 |
| BP2 | 3 | 0.81 |
| Affect_somatic | 3 | 0.81 |
| Cannabis | 3 | 0.81 |
| Anx_somatic | 2 | 0.54 |
| Schizoaffective | 1 | 0.27 |
| Psychosis_IEC | 1 | 0.27 |
| Sedative | 1 | 0.27 |
| BPother | 0 | 0.00 |
| Affect_substance | 0 | 0.00 |
| Schizophrenia | 0 | 0.00 |
| Schizophreniform | 0 | 0.00 |
| Paranoid | 0 | 0.00 |
| Psychosis | 0 | 0.00 |
| Psychosis_somatic | 0 | 0.00 |
| Psychosis_substance | 0 | 0.00 |
| Stimulant | 0 | 0.00 |
| Opiate | 0 | 0.00 |
| Cocaine | 0 | 0.00 |
| PCP | 0 | 0.00 |
| PolySubstance | 0 | 0.00 |
| OtherSubstance | 0 | 0.00 |
| Anx_substance | 0 | 0.00 |
| *Note:* DSM-IV diagnoses. Percentage out of all 370 patients. | | |
| **Table** | | |
| *Frequency of DSM-IV Axis II Disorders at T1* | | |
| PD | Frequency | % |
| depressive | 102 | 27.57 |
| avoidant | 98 | 26.49 |
| obsessive | 54 | 14.59 |
| paranoid | 46 | 12.43 |
| borderline | 32 | 8.65 |
| dependent | 21 | 5.68 |
| negativistic | 19 | 5.14 |
| antisocial | 9 | 2.43 |
| schizoid | 8 | 2.16 |
| NOS | 7 | 1.89 |
| schizotype | 6 | 1.62 |
| histrionic | 1 | 0.27 |
| narsissistic | 1 | 0.27 |
| *Note:* DSM-IV Personality Disorders. Percentage out of all 370 patients. | | |

| **Table** | | |
| --- | --- | --- |
| *Quantity and Quality of Social Relationships Indexes Cronbach's Alpha Values at T1* | | |
| Index | Raw alpha | Standardized alpha |
| Total | 0.90 | 0.90 |
| Contact | 0.68 | 0.67 |
| Faffect | 0.80 | 0.80 |
| Raffect | 0.78 | 0.78 |
| Support | 0.76 | 0.76 |
| FamOri | 0.90 | 0.90 |
| CurFam | 0.64 | 0.64 |
| OutFam | 0.86 | 0.87 |
| NQQSR | 0.34 | 0.38 |
| NFamOri | 0.40 | 0.36 |
| NCurFam | 0.45 | 0.45 |
| NOutFam | 0.31 | 0.32 |
