# Supplementary material for: Connected to Others and Kind to Self: Social Connection and Self‐Affiliation Change in Open‐Ended Psychotherapy
Source: J Clin Psychol. 2026 Sep 4;82(12):2060–9. doi: 10.1002/jclp.70220 (PMC13733646; doi:10.1002/jclp.70220)
Supplement: Supplementary file 4 [file JCLP-82-2060-s004.docx]

### Supplement 03

### Univariate Latent Curve Model Results for the Quantity and Quality of Social Relationships

| *Fit Statistics: Latent Curve Models of QQSR T1–T5* | | | | | | | |
| --- | --- | --- | --- | --- | --- | --- | --- |
| Variable | CFI | TLI | SRMR | χ^2^ | *df* | *p* | RMSEA [90% CI] |
| TotalQQSR | 1.0 | 1.0 | .03 | 12.95 | 10 | .226 | 0.03 [0.00, 0.07] |
| Contact | .97 | .97 | .05 | 33.87 | 10 | < .001*** | 0.08 [0.05, 0.11] |
| Faffect | 1.0 | 1.0 | .03 | 8.93 | 10 | .539 | 0.00 [0.00, 0.05] |
| Raffect | .99 | .99 | .04 | 15.39 | 10 | .118 | 0.04 [0.00, 0.07] |
| Support | .99 | .99 | .04 | 16.00 | 10 | .100 | 0.04 [0.00, 0.08] |
| FamOri | 1.0 | 1.0 | .03 | 6.28 | 10 | .791 | 0.00 [0.00, 0.04] |
| CurFam | .97 | .97 | .07 | 22.21 | 10 | .014* | 0.06 [0.03, 0.09] |
| OutFam | 1.0 | 1.0 | .04 | 8.90 | 10 | .542 | 0.00 [0.00, 0.05] |
| NQQSR | .97 | .97 | .07 | 28.97 | 10 | .001** | 0.07 [0.04, 0.11] |
| NFamOri | .98 | .98 | .05 | 36.08 | 10 | < .001*** | 0.08 [0.06, 0.12] |
| NCurFam | .98 | .98 | .04 | 29.89 | 10 | .001*** | 0.07 [0.04, 0.10] |
| NOutFam | .96 | .96 | .07 | 22.86 | 10 | .011* | 0.06 [0.03, 0.09] |
| *Note.* Random slopes latent growth curve models. CFI = Comparative Fit Index, TLI = Tucker-Lewis Index, SRMR = Standardized Root Mean Square Residual, RMSEA = Root Mean Square Error of Approximation with 90% Confidence Interval, Chi-square statistic with degrees of freedom and p-value comparing the model to a saturated model. * = p < .05, ** = p < .01, *** = p <.001. QQSR = Quantity and Quality of Social Relationships, Contact = frequency of contact, Faffect = freely expressed affection, Raffect = received affection, FamOri = family of origin (mother, father, sibling, other relation), CurFam = current family (partner and child), OutFam = outside family (friend, colleagues, neighbours). N = number of relationships present. t1 = pretreatment, t2 = post treatment, t3 = six months post t2, t4 = one year post t2, t5 = two and a half years post t2. * = p < .05, ** = p < .01, *** = p < .001. | | | | | | | |

| *Fixed Effects of Latent Curve Models of QQSR T1–T5* | | | | | |
| --- | --- | --- | --- | --- | --- |
| Index | Parameter | *SE* | *Z* | *p* | b [95% CI] |
| TotalQQSR | Intercept | 0.02 | 115.41 | < .001*** | 2.69 [2.64, 2.73] |
|  | Slope | 0.01 | 2.95 | .003** | 0.02 [0.01, 0.03] |
| Contact | Intercept | 0.04 | 109.81 | < .001*** | 4.31 [4.23, 4.38] |
|  | Slope | 0.01 | 1.29 | .196 | 0.01 [-0.01, 0.04] |
| Faffect | Intercept | 0.04 | 83.54 | < .001*** | 3.26 [3.19, 3.34] |
|  | Slope | 0.01 | 2.09 | .036* | 0.02 [0.00, 0.04] |
| Raffect | Intercept | 0.04 | 81.53 | < .001*** | 3.28 [3.20, 3.36] |
|  | Slope | 0.01 | 2.00 | .045* | 0.02 [0.00, 0.04] |
| Support | Intercept | 0.03 | 76.49 | < .001*** | 2.37 [2.31, 2.43] |
|  | Slope | 0.01 | 3.26 | .001** | 0.03 [0.01, 0.04] |
| FamOri | Intercept | 0.03 | 77.54 | < .001*** | 2.51 [2.45, 2.58] |
|  | Slope | 0.01 | 0.69 | .489 | 0.01 [-0.01, 0.02] |
| CurFam | Intercept | 0.03 | 127.44 | < .001*** | 3.48 [3.42, 3.53] |
|  | Slope | 0.01 | 1.70 | .089 | 0.01 [-0.00, 0.02] |
| OutFam | Intercept | 0.03 | 82.89 | < .001*** | 2.49 [2.43, 2.55] |
|  | Slope | 0.01 | 3.03 | .002** | 0.03 [0.01, 0.04] |
| NQQSR | Intercept | 0.07 | 115.37 | < .001*** | 8.29 [8.15, 8.43] |
|  | Slope | 0.02 | -1.23 | .220 | -0.02 [-0.06, 0.01] |
| NFamOri | Intercept | 0.04 | 80.58 | < .001*** | 3.43 [3.34, 3.51] |
|  | Slope | 0.01 | -4.90 | < .001*** | -0.05 [-0.07, -0.03] |
| NCurFam | Intercept | 0.04 | 35.65 | < .001*** | 1.44 [1.36, 1.52] |
|  | Slope | 0.01 | 2.47 | .014* | 0.02 [0.00, 0.03] |
| NOutFam | Intercept | 0.04 | 86.00 | < .001*** | 3.44 [3.36, 3.51] |
|  | Slope | 0.01 | 0.53 | .593 | 0.01 [-0.02, 0.03] |
| *Note.* Effect of time as years in random slopes latent growth curve models. Cluster-robust standard errors for therapist-level clustering. QQSR = Quantity and Quality of Social Relationships, Contact = frequency of contact, Faffect = freely expressed affection, Raffect = received affection, FamOri = family of origin (mother, father, sibling, other relation), CurFam = current family (partner and child), OutFam = outside family (friend, colleagues, neighbours). N = number of relationships present. t1 = pretreatment, t2 = post treatment, t3 = six months post t2, t4 = one year post t2, t5 = two and a half years post t2. * = p < .05, ** = p < .01, *** = p < .001. | | | | | |

| *Random Effects Variance and Covariance of Latent Curve Models of QQSR T1–T5* | | | | | |
| --- | --- | --- | --- | --- | --- |
| Index | Parameter | Var | *SE* | *Z* | *p* |
| TotalQQSR | Intercept variance | 0.14 | 0.01 | 11.17 | < .001*** |
| TotalQQSR | Slope variance | 0.01 | 0.00 | 3.11 | .002** |
| TotalQQSR | Int-Slope covariance | -0.00 | 0.00 | -1.23 | .220 |
| Contact | Intercept variance | 0.39 | 0.04 | 9.53 | < .001*** |
| Contact | Slope variance | 0.02 | 0.00 | 3.83 | < .001*** |
| Contact | Int-Slope covariance | -0.04 | 0.01 | -2.87 | .004** |
| Faffect | Intercept variance | 0.39 | 0.04 | 10.87 | < .001*** |
| Faffect | Slope variance | 0.01 | 0.01 | 2.53 | .011* |
| Faffect | Int-Slope covariance | -0.02 | 0.01 | -1.82 | .069 |
| Raffect | Intercept variance | 0.37 | 0.04 | 8.90 | < .001*** |
| Raffect | Slope variance | 0.01 | 0.00 | 2.06 | .039* |
| Raffect | Int-Slope covariance | -0.01 | 0.01 | -1.08 | .278 |
| Support | Intercept variance | 0.19 | 0.02 | 8.74 | < .001*** |
| Support | Slope variance | 0.01 | 0.00 | 2.97 | .003** |
| Support | Int-Slope covariance | 0.00 | 0.01 | 0.57 | .570 |
| FamOri | Intercept variance | 0.30 | 0.02 | 12.19 | < .001*** |
| FamOri | Slope variance | 0.01 | 0.00 | 1.65 | .098 |
| FamOri | Int-Slope covariance | -0.00 | 0.01 | -0.14 | .886 |
| CurFam | Intercept variance | 0.14 | 0.04 | 3.39 | .001*** |
| CurFam | Slope variance | 0.01 | 0.00 | 2.22 | .026* |
| CurFam | Int-Slope covariance | -0.01 | 0.01 | -0.74 | .461 |
| OutFam | Intercept variance | 0.20 | 0.02 | 8.82 | < .001*** |
| OutFam | Slope variance | 0.01 | 0.00 | 3.27 | .001** |
| OutFam | Int-Slope covariance | -0.01 | 0.01 | -1.20 | .231 |
| NQQSR | Intercept variance | 1.51 | 0.19 | 7.85 | < .001*** |
| NQQSR | Slope variance | 0.04 | 0.02 | 2.23 | .025* |
| NQQSR | Int-Slope covariance | -0.03 | 0.04 | -0.77 | .441 |
| NFamOri | Intercept variance | 0.55 | 0.05 | 10.32 | < .001*** |
| NFamOri | Slope variance | 0.00 | 0.01 | 0.56 | .578 |
| NFamOri | Int-Slope covariance | 0.01 | 0.02 | 0.82 | .414 |
| NCurFam | Intercept variance | 0.43 | 0.03 | 12.79 | < .001*** |
| NCurFam | Slope variance | 0.01 | 0.00 | 1.65 | .099 |
| NCurFam | Int-Slope covariance | -0.01 | 0.01 | -1.29 | .198 |
| NOutFam | Intercept variance | 0.30 | 0.06 | 5.06 | < .001*** |
| NOutFam | Slope variance | 0.01 | 0.01 | 1.52 | .129 |
| NOutFam | Int-Slope covariance | -0.01 | 0.02 | -0.60 | .550 |
| *Note.* Random slopes latent growth curve models. QQSR = Quantity and Quality of Social Relationships, Contact = frequency of contact, Faffect = freely expressed affection, Raffect = received affection, FamOri = family of origin (mother, father, sibling, other relation), CurFam = current family (partner and child), OutFam = outside family (friend, colleagues, neighbours). N = number of relationships present. t1 = pretreatment, t2 = post treatment, t3 = six months post t2, t4 = one year post t2, t5 = two and a half years post t2. * = p < .05, ** = p < .01, *** = p < .001. | | | | | |

| *Fixed Effects of Latent Curve Models of QQSR T1–T2* | | | | | |
| --- | --- | --- | --- | --- | --- |
| Index | Parameter | *SE* | *Z* | *p* | b [95% CI] |
| TotalQQSR | Intercept | 0.03 | 105.00 | < .001*** | 2.68 [2.63, 2.73] |
|  | Slope | 0.02 | 1.18 | .239 | 0.03 [-0.02, 0.07] |
| Contact | Intercept | 0.04 | 102.71 | < .001*** | 4.29 [4.21, 4.38] |
|  | Slope | 0.03 | 1.16 | .246 | 0.04 [-0.03, 0.11] |
| Faffect | Intercept | 0.04 | 78.80 | < .001*** | 3.25 [3.17, 3.33] |
|  | Slope | 0.04 | 1.11 | .268 | 0.05 [-0.04, 0.13] |
| Raffect | Intercept | 0.04 | 75.18 | < .001*** | 3.29 [3.21, 3.38] |
|  | Slope | 0.04 | 0.09 | .926 | 0.00 [-0.07, 0.08] |
| Support | Intercept | 0.03 | 70.27 | < .001*** | 2.36 [2.30, 2.43] |
|  | Slope | 0.03 | 1.57 | .116 | 0.05 [-0.01, 0.12] |
| FamOri | Intercept | 0.04 | 69.55 | < .001*** | 2.51 [2.44, 2.58] |
|  | Slope | 0.03 | 0.36 | .722 | 0.01 [-0.04, 0.06] |
| CurFam | Intercept | 0.03 | 128.81 | < .001*** | 3.47 [3.42, 3.52] |
|  | Slope | 0.03 | 0.13 | .893 | 0.00 [-0.06, 0.07] |
| OutFam | Intercept | 0.03 | 77.47 | < .001*** | 2.48 [2.42, 2.55] |
|  | Slope | 0.03 | 1.35 | .176 | 0.04 [-0.02, 0.11] |
| NQQSR | Intercept | 0.08 | 107.70 | < .001*** | 8.31 [8.16, 8.46] |
|  | Slope | 0.08 | -0.71 | .478 | -0.06 [-0.22, 0.10] |
| NFamOri | Intercept | 0.04 | 82.12 | < .001*** | 3.45 [3.37, 3.53] |
|  | Slope | 0.03 | -3.13 | .002** | -0.10 [-0.16, -0.04] |
| NCurFam | Intercept | 0.04 | 34.25 | < .001*** | 1.43 [1.35, 1.52] |
|  | Slope | 0.03 | 2.56 | .011* | 0.07 [0.02, 0.12] |
| NOutFam | Intercept | 0.04 | 84.22 | < .001*** | 3.45 [3.37, 3.54] |
|  | Slope | 0.06 | -0.33 | .743 | -0.02 [-0.13, 0.10] |
| *Note.* Difference from T1 to T2 in random intercept latent growth curve models. Cluster-robust standard errors for therapist-level clustering. QQSR = Quantity and Quality of Social Relationships, Contact = frequency of contact, Faffect = freely expressed affection, Raffect = received affection, FamOri = family of origin (mother, father, sibling, other relation), CurFam = current family (partner and child), OutFam = outside family (friend, colleagues, neighbours). N = number of relationships present. t1 = pretreatment, t2 = post treatment, t3 = six months post t2, t4 = one year post t2, t5 = two and a half years post t2. * = p < .05, ** = p < .01, *** = p < .001. | | | | | |
